# ε‐Poly‐L‐lysine‐*graft*‐oligo(3‐hexylthiophene) Copolymers as Antibacterial and Biodegradable Polymer Electronics

**DOI:** 10.1002/advs.202501726

**Published:** 2025-05-28

**Authors:** Xin Sun, Eddie Wai Chi Chan, Fathumma Rizana Shiraz, Bicheng Zhu, Jingwen Yang, Katharina Matura, Viji Sarojini, Serpil Tekoglu, David Barker, Jadranka Travas‐Sejdic

**Affiliations:** ^1^ Centre for Innovative Materials for Health School of Chemical Sciences The University of Auckland Auckland 1010 New Zealand; ^2^ MacDiarmid Institute for Advanced Materials and Nanotechnology Wellington 6140 New Zealand; ^3^ School of Chemical Sciences and The Centre for Green Chemical Science University of Auckland Auckland 1142 New Zealand; ^4^ Linz Institute for Organic Solar Cells and Institute of Physical Chemistry Johannes Kepler University Linz Altenbergerstrasse 69 Linz 4040 Austria

**Keywords:** antibacterial ability, biodegradable polymer electronics, biopolymers, conducting polymers, multifunctional electronics

## Abstract

Biodegradable polymer electronics offer an innovative solution to the growing challenge of electronic waste, which are engineered to disintegrate after a defined functional period. Here, a new class of graft copolymer is presented, ε‐poly‐L‐lysine‐*graft*‐oligo(3‐hexylthiophene) (EPL‐*g*‐O3HTs), synthesized by covalently grafting oligo(3‐hexylthiophene) onto the biopolymer ε‐poly‐L‐lysine at three grafting densities, resulting in copolymers containing 43, 65 and 90 wt.% O3HT (EPL‐*g*‐O3HT‐1, EPL‐*g*‐O3HT‐2 and EPL‐*g*‐O3HT‐3, respectively). Benefiting from the “guidance” of ε‐poly‐L‐lysine on O3HT chains alignment, the graft copolymer with optimized grafting density exhibits an extended conjugation length and increased crystallite size of O3HT. Thin films of three copolymers, upon doping, demonstrate appreciable conductivity under ambient conditions. EPL‐*g*‐O3HT‐1 could be fully break down over 12 days by enzymatic degradation. EPL‐*g*‐O3HT‐1 also displays excellent broad‐spectrum antibacterial activity against Gram‐negative and Gram‐positive bacteria, attributed to its high ɛ‐poly‐L‐lysine content. It is further demonstrated the versatility of EPL‐*g*‐O3HTs in transient electronics for electromyography sensors for muscle signal acquisition and as the channel material in organic electrochemical transistors. Combining tunable conductivity, controlled biodegradability, and antimicrobial properties, EPL‐*g*‐O3HT copolymers hold significant potential for diverse transient electronic applications, including skin and implantable electronics, where degradable electronics with antimicrobial properties are highly desirable.

## Graphical abstract

ε‐Poly‐L‐lysine‐*graft*‐oligo(3‐hexylthiophene) (EPL‐*g*‐O3HT) copolymers are engineered as biodegradable and antibacterial materials for transient electronics. EPL‐*g*‐O3HT exhibits tunable conductivity, controlled enzymatic degradability, good biocompatibility, and potent antibacterial activity. They are demonstrated in electromyography sensors and organic electrochemical transistors. EPL‐*g*‐O3HT copolymers hold significant potential for transient electronic applications, including skin and implantable electronics, where degradable electronics with antimicrobial properties are highly desirable.

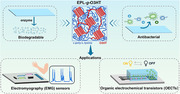

## 1 Introduction

The rapid growth of consumer electronics has led to a significant increase in electronic waste, posing a major environmental challenge.^[^ ^1^ ^]^ Transient electronics offer a novel and sustainable approach to addressing this issue by providing devices that disintegrate safely after their functional lifetime, thereby reducing or eliminating the burden of e‐waste on society.^[^ ^2^ ^]^ A promising strategy for designing such systems is to graft conducting polymers onto biodegradable polymer backbone through covalent grafting.^[^ ^3^ ^]^ This molecular‐level integration enables the simultaneous realization of electrical functionality and controlled degradation.

In biodegradable graft copolymers, the backbone polymer serves as a structural guide for the covalently grafted conducting polymer, enabling the formation of well‐ordered crystallites or interconnected crystalline domains. This approach is different from blends or composites, where a conducting polymer and a degradable polymer are merely physically mixed.^[^ ^3^, ^4^ ^]^ These interconnected domains facilitate efficient charge transfer, thereby enhancing the conductivity of the material.^[^ ^4a,b^ ^]^ Furthermore, the degradation behavior of the material can be inherited from biodegradable backbone polymers that contain cleavage points, affording a material with both electrical and transient properties within a single macromolecular.^[^ ^4^, ^5^ ^]^

Incorporating antibacterial functionality into biodegradable electronics is also highly desirable, particularly for applications involving skin contact or implantation, where bacterial colonization at the device–tissue interface may cause infection or inflammation.^[^ ^6^ ^]^ Biopolymers present an ideal choice for the polymer backbone, owing to their inherent biodegradability, biocompatibility, and, in some cases, additional properties such as antibacterial activity.^[^ ^3^, ^7^ ^]^ Our previous work demonstrated the feasibility of combining conducting polymers with biopolymers as transient pressure sensors, by grafting poly(3‐hexylthiophene) onto gelatin to create gelatin‐*graft*‐poly(3‐hexylthiophene).^[^ ^5a^ ^]^ In this context, biopolymer ε‐poly‐L‐lysine (EPL), which possesses inherent antibacterial properties and biodegradability, emerges as a particularly promising backbone polymer for the development of biodegradable electronics with integrated antibacterial functionality.^[^ ^8^ ^]^

In this study, we synthesized a new class of graft copolymers by covalently attaching oligo(3‐hexylthiophene) (O3HT‐NHS) to ε‐poly‐L‐lysine (EPL) at varying grafting densities. The EPL‐*graft*‐O3HT (EPL‐*g*‐O3HTs) with 43, 65 and 90 wt.% of O3HT grafted (EPL‐*g*‐O3HT‐1, EPL‐*g*‐O3HT‐2 and EPL‐*g*‐O3HT‐3, respectively) exhibit distinct properties influenced by the amount of O3HT grafted. Among these, thin films of EPL‐*g*‐O3HT‐1 exhibited both significant conductivity (1.13 ± 0.03 S m^−1^) and controllable degradation behavior, extensively disintegrating under trypsin‐mediated enzymatic degradation over 12 days. It also displayed broad‐spectrum antibacterial activity at a concentration of 12 mg mL^−1^, achieving 97% and 89.6% bactericidal efficacy against *Escherichia coli* and *Staphylococcus aureus* bacteria, respectively. The thin films of EPL‐*g*‐O3HT‐1 were validated in an electromyography (EMG) sensor to measure muscle activity in real‐time and as the channel material in organic electrochemical transistors (OECTs) device that displays a significant on‐to‐off current ratio. These results highlight the significant potential of EPL‐*g*‐O3HT copolymers as versatile and functional active components in a wide range of transient polymer electronics applications, leveraging macromolecular design that integrates conductivity, degradability and antimicrobial properties.

## 2 Results and Discussion

### 2.1 Synthesis and Characterizations of EPL‐g‐O3HTs

**Scheme 1:**
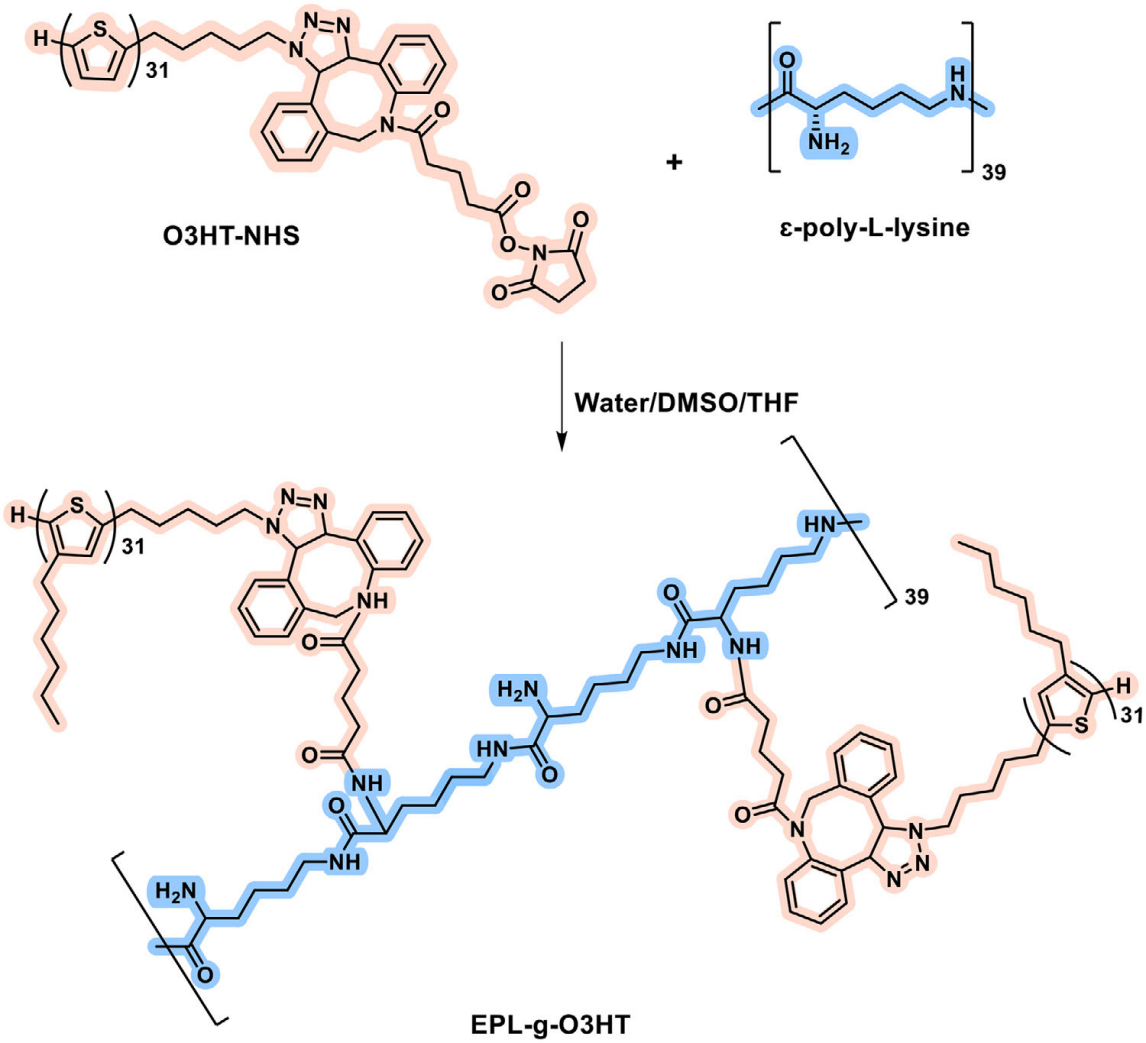
Synthesis of the copolymer EPL‐*g*‐O3HTs by grafting of O3HT onto ɛ‐poly‐L‐lysine backbone via amide bonds.

**Figure 1:**
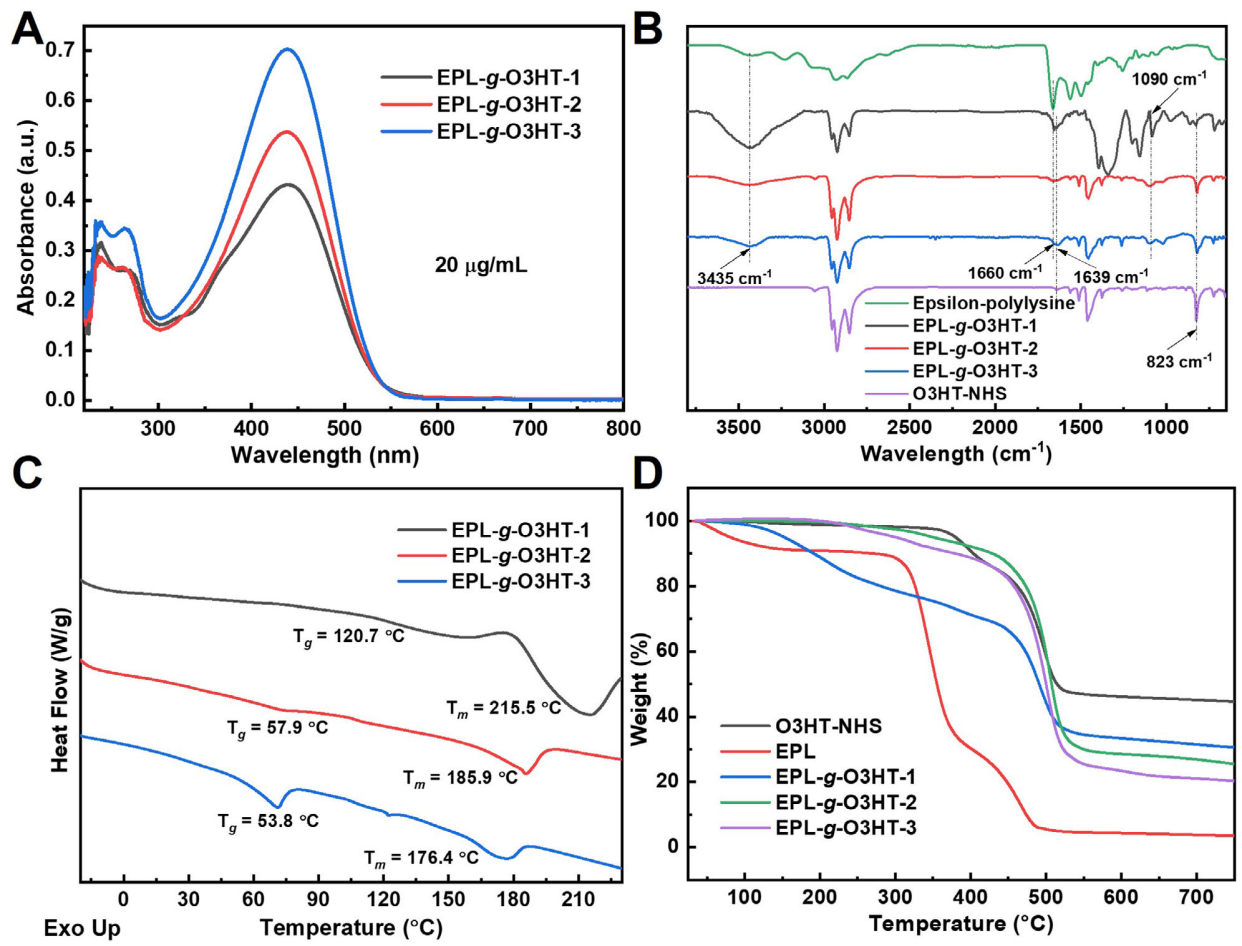
Characterizations of EPL‐*g*‐O3HTs. A) UV–vis spectra of EPL‐*g*‐O3HTs copolymers at a concentration of 20 µg mL^−1^. B) FTIR spectra of ɛ‐poly‐L‐lysine, O3HT‐NHS and EPL‐*g*‐O3HTs. C) DSC scans of EPL‐*g*‐O3HTs. D) TGA curves of the ɛ‐poly‐L‐lysine, O3HT‐NHS and EPL‐*g*‐O3HTs.

EPL‐*g*‐O3HT was successfully synthesized by reacting O3HT‐NHS with ɛ‐poly‐L‐lysine, allowing the reaction to proceed overnight. In the resulting EPL‐*g*‐O3HT copolymers (**Scheme** **1**), ε‐poly‐L‐lysine serves as the degradable backbone, while O3HT acts as the conductive component. By varying the feed ratio of O3HT‐NHS, three copolymers, EPL‐*g*‐O3HT‐1, EPL‐*g*‐O3HT‐2, and EPL‐*g*‐O3HT‐3, were synthesized, containing 43, 65, and 90 wt.% O3HT, respectively. The wt.% of O3HT‐NHS in EPL‐*g*‐O3HTs was determined using the calibration curve of O3HT‐NHS in THF obtained by UV–vis spectroscopy (Figure S1, Supporting Information).^[^ ^5a^ ^]^ The UV–vis spectra of the EPL‐*g*‐O3HTs, presented in **Figure** **1**A, feature a prominent peak at ≈440 nm, corresponding to the π–π* transition of O3HT within the EPL‐*g*‐O3HT copolymers. Elemental analysis was further employed to quantitatively confirm the grafting density. As shown in Table S1 (Supporting Information), the S/N weight ratio gradually increased from 9.4 for EPL‐*g*‐O3HT‐1), to 11.8 for EPL‐*g*‐O3HT‐2, and 24.2 for EPL‐*g*‐O3HT‐3, as the grafting density rose from 43% to 65% and 90%, respectively. With N atoms introduced by the NHS linker in O3HT‐NHS, the S/N weight ratio was 40.4. These results further support the successful grafting of O3HT onto EPL.

FTIR and NMR spectroscopies were utilized to characterize the chemical structure of EPL‐*g*‐O3HTs. The FTIR spectra in Figure 1B confirm the successful synthesis of EPL‐*g*‐O3HTs. The peak at 1090 cm^−1^ in EPL‐*g*‐O3HTs corresponds to C‐N stretching, confirming the presence of amide bonds within the EPL component of EPL‐*g*‐O3HTs.^[^ ^9^ ^]^ The broad absorption band at 3435 cm^−1^ is associated with N─H stretching, and its higher intensity compared to EPL could be due to intermolecular hydrogen bonding between the C ═ O of O3HT‐NHS and the amine groups of EPL.^[^ ^10^ ^]^ Characteristic peaks at 1660 and 1639 cm^−1^ are attributed to the C ═ O stretching of O3HT^[^ ^11^ ^]^ and ε‐poly‐L‐lysine,^[^ ^9^ ^]^ respectively, with their intensities varying according to the grafting density. Specifically, in EPL‐*g*‐O3HT‐1, the C ═ O stretching from ε‐poly‐L‐lysine PL shows a higher intensity than that of O3HT, whereas, in EPL‐*g*‐O3HT‐3, the peak intensities are reversed, with O3HT dominating. In EPL‐*g*‐O3HT‐2, the C ═ O stretching from both components exhibits comparable intensities. Additionally, EPL‐*g*‐O3HTs exhibit a peak at 823 cm^−1^, which intensifies with increasing O3HT content, further supporting the presence of O3HT in the graft copolymer. In the NMR spectra (Figure S2A,B, Supporting Information), a new peak appeared ≈3.7 ppm, corresponding to the ‐CH_2_ group adjacent to the NH_2_ groups in the side chains of EPL.^[^ ^12^ ^]^ In EPL‐*g*‐O3HT‐3, no new peak is observed, which suggests that a significant portion of the amine groups have reacted with O3HT, leading to the disappearance of the peak at 3.7 ppm. These findings conclusively demonstrate the successful synthesis of the desired EPL‐*g*‐O3HT graft copolymers.

Thermal properties of the EPL‐*g*‐O3HTs graft copolymers were evaluated using DSC and TGA. The DSC traces (Figure 1C) indicate the glass transition temperatures (*T*_g_) of EPL‐*g*‐O3HT‐1, EPL‐*g*‐O3HT‐2 and EPL‐*g*‐O3HT‐3 as 120.7, 57.9 and 53.8 °C, respectively, with corresponding melting temperatures (*T*_m_) of 215.5, 185.9 and 176.4 °C. As the O3HT content increases, both *T*_g_ and *T*_m_ decrease, gradually approaching the values of the O3HT homopolymers (O3HT‐NHS: *T_g_* = 57.5 °C, *T_m_* = 150.3 °C), as shown in Figure S3A (Supporting Information). Notably, the *T*_g_ of EPL‐*g*‐O3HT‐1 and the *T*_m_ of all EPL‐*g*‐O3HT copolymers are higher than those of both O3HT‐NHS and ε‐poly‐L‐lysine (*T_g_* = 86.7 °C, *T_m_* = 146.2 °C, shown in Figure S3B, Supporting Information). This can be attributed to the intermolecular hydrogen bonding between the C ═ O groups of O3HT and the amine groups of ɛ‐poly‐L‐lysine. Such interactions likely reduce chain segment mobility and decrease free volume within the graft copolymer, resulting in a higher *T_g_*.^[^ ^13^ ^]^ The enhanced intermolecular hydrogen bonding also contributes to an increased *T_m_*, reflecting enhanced thermal stability.^[^ ^14^ ^]^ TGA and differential thermogravimetric analysis (DGA) curves (Figure 1D; Figure S4, Supporting Information) reveal that the first weight loss of EPL‐*g*‐O3HTs copolymers starts at ≈200 °C, with a second significant weight loss occurring between 400 and 600 °C. The initial weight loss is attributed to the degradation of the EPL component, while the second weight loss corresponds to the degradation of the O3HT segments, as indicated by the TGA and DGA curves of EPL and O3HT‐NHS (Figure 1D; Figure S4, Supporting Information). The early weight loss of EPL observed before 50 °C corresponds to the loss of adsorbed water.^[^ ^8a^ ^]^ These thermal analysis results further support the successful synthesis of EPL‐*g*‐O3HTs.

### 2.2 Structural Characterizations of EPL‐g‐O3HTs Films

**Figure 2:**
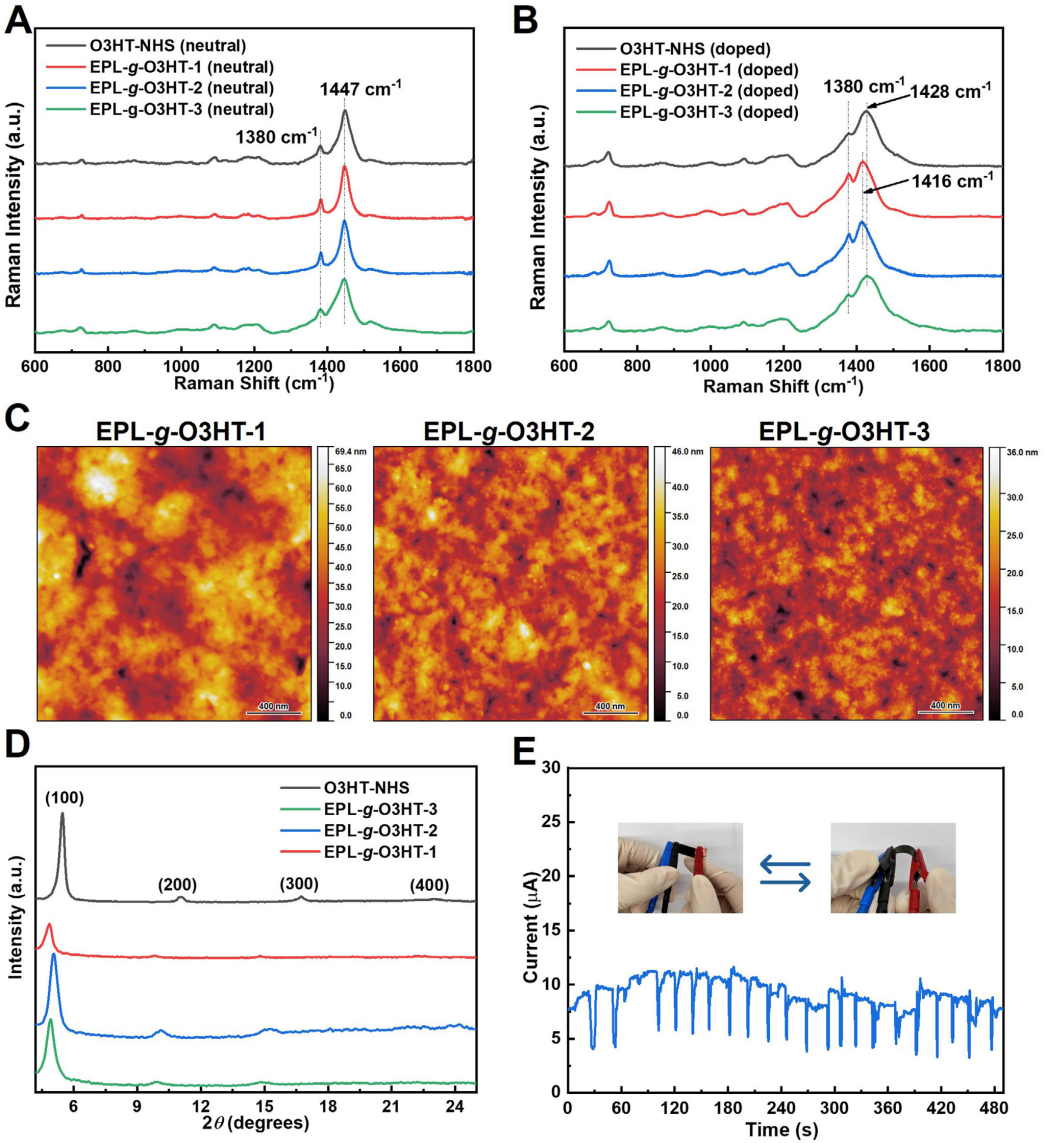
Structural characterizations of EPL‐*g*‐O3HTs. Raman spectra of O3HT‐NHS and EPL‐*g*‐O3HTs in neutral state A) and doped state B). C) AFM height images of O3HT‐NHS and EPL‐*g*‐O3HTs (doped). D) XRD spectra of O3HT‐NHS and EPL‐*g*‐O3HTs (doped). FeCl_3_ was employed as the dopant. E) Cyclic bending test of EPL‐*g*‐O3HT‐1 thin film on PET substrate, with the current continuously monitored throughout the bending cycles.

To investigate the thin film structure of EPL‐*g*‐O3HTs, Raman spectroscopy was employed for detailed characterizations. The Raman spectra of both neutral state and doped EPL‐*g*‐O3HTs thin films, excited at 785 nm, are shown in **Figure** **2**A,B. In the neutral state, EPL‐*g*‐O3HTs thin films (Figure 2A) present several characteristic Raman modes of O3HT,^[^ ^15^ ^]^ with no Raman modes of EPL visible (Figure S5, Supporting Information). The two prominent diagnostic peaks at 1447 and 1380 cm^−1^ are attributed to the C ═ C symmetric stretch mode and the C─C intra‐ring stretch mode, respectively, related to in‐plane vibrations within the thiophene ring of the O3HT polymer backbone. Additionally, the peak at 728 cm^−1^ corresponds to the C─S─C deformation mode, while the peak ≈1210 cm^−1^ represents the inter‐ring C─C stretch mode. Among these Raman modes of O3HT, we focus on the two prominent diagnostic in‐plane peaks: C ═ C symmetric stretch mode and C─C intra‐ring stretch mode, as they are sensitive to the conjugation length of O3HT molecules.^[^ ^15^, ^16^ ^]^ Upon doping the EPL‐*g*‐O3HTs thin films (Figure 2B), a significant spectral shift is observed: the C ═ C peak moves from 1447 cm^−1^ to a lower wavenumber (1416/1428 cm^−1^), in line with the effect of FeCl_3_ doping in P3HT.^[^ ^11^ ^]^ This shift reflects the formation of polarons in doped O3HT, as the C ═ C bonds within the O3HT thiophene rings extend in length and exhibit more C─C‐like characteristics due to a decrease in electron density.^[^ ^15^, ^17^ ^]^ With the decreased electron density, the C ═ C double bonds require less energy (corresponding to a lower wavenumber) to undergo stretching vibrations. Notably, the C ═ C symmetric stretch modes of EPL‐*g*‐O3HT‐1 and EPL‐*g*‐O3HT‐2 shift further downward to 1416 cm^−1^, which is lower than that observed in O3HT‐NHS and EPL‐*g*‐O3HT‐3 (1428 cm^−1^).

In addition, Tsoi et al. have showed that a higher intensity ratio of the C─C intra‐ring stretch mode to the C ═ C symmetric stretch mode (I*_C─C_*/I*_C ═ C_*) is associated with increased backbone planarity in oligothiophene.^[^ ^28^ ^]^ The more planar O3HT backbone promotes π‐stacking among polymer chains, leading to an extended conjugation length and enhanced O3HT molecular ordering.^[^ ^18^ ^]^ *I_C─C_*/I*_C ═ C_* values of neutral and doped samples are shown in Table S2 (Supporting Information). All doped EPL‐*g*‐O3HTs and O3HT‐NHS thin films exhibit increased I*_C─C_*/I*_C ═ C_* ratios compared to their neutral states, suggesting that doping enhances O3HT molecular ordering after doping. Specifically, EPL‐*g*‐O3HT‐1 (I*_C─C_*/I*_C ═ C_* = 0.76) and EPL‐*g*‐O3HT‐2 (I*_C─C_*/I*_C ═ C_* = 0.75) have notably higher I*_C─C_*/I*_C ═ C_* ratios than O3HT‐NHS (I*_C─C_*/I*_C ═ C_* = 0.60) and EPL‐*g*‐O3HT‐3 (I*_C─C_*/I*_C ═ C_* = 0.53). The similarity of EPL‐*g*‐O3HT‐3′s ratio to O3HT‐NHS is likely due to its low EPL content (10 wt.% relative to 90% of O3HT). The peak shifts of the C ═ C symmetric stretch mode to a lower wavenumber and the higher I*_C─C_*/I*_C ═ C_* ratios observed in EPL‐*g*‐O3HT‐1 and EPL‐*g*‐O3HT‐2, suggest an extended conjugation length and increased molecular ordering of O3HT compared to pristine O3HT‐NHS, which could be attributed to guidance of EPL backbone after O3HT grafting and are consistent with findings from our previous study on poly(caprolactone‐*co*‐3‐alkyne‐valerolactone)‐*graft*‐oligo‐3‐hexylthiophene.^[^ ^15b^ ^]^

The thin film morphology and crystallinity of EPL‐*g*‐O3HTs graft copolymers were further evaluated by atomic force microscopy (AFM) and powder X‐ray diffraction (XRD). In the height images of AFM (Figure 2C), EPL‐*g*‐O3HT‐1 exhibited a root mean square (RMS) roughness of 18.96 nm, significantly higher than EPL‐*g*‐O3HT‐2 (3.55 nm) and EPL‐*g*‐O3HT‐3 (3.90 nm). This increased roughness for EPL‐*g*‐O3HT‐1 film might be attributed to the larger amount of EPL present, which is immiscible with O3HT in the processing solvent THF. XRD spectra of EPL‐*g*‐O3HTs were collected in the 2*θ* ranges of 4° to 25° (Figure 2D). Compared to O3HT‐NHS, thin films of EPL‐*g*‐O3HTs exhibit attenuated multi‐order (*h*00) reflections. Multiple (*h*00) reflections represent the lamellar spacing of O3HT, which corresponds to the distance between the polymer chains in the crystalline lamellar structure.^[^ ^15b^ ^]^ The (100) reflection for EPL‐*g*‐O3HT‐1, EPL‐*g*‐O3HT‐2 and EPL‐*g*‐O3HT‐3 appears at 2*θ* values of 4.87°, 5.04°, and 4.90°, respectively, corresponding to the d‐spacing of 18.13, 17.52, and 18.02 Å. These d‐spacing values are consistently larger than that of pristine O3HT‐NHS (16.35 Å),^[^ ^15b^ ^]^ which may be ascribed to O3HTs being grafted onto EPL, with grafting disrupting the tight packing of O3HT chains.^[^ ^15^, ^19^ ^]^ Additionally, the crystallite size along the vertical direction, L, was calculated using the Scherrer equation from the full width at half maximum (FWHM) of the primary (100) peak.^[^ ^20^ ^]^ The crystallite sizes for EPL‐*g*‐O3HT‐1, EPL‐*g*‐O3HT‐2 and EPL‐*g*‐O3HT‐3 were calculated to be 129.5 nm (FWHM: 0.61), 156.2 nm (FWHM: 0.51) and 129.0 nm (FWHM: 0.62), respectively. Notably, EPL‐*g*‐O3HT‐1 has a crystallite size similar to EPL‐*g*‐O3HT‐3, while EPL‐*g*‐O3HT‐2 even exhibits a larger crystallite size than EPL‐*g*‐O3HT‐3. This unexpectedly large crystallite size in EPL‐*g*‐O3HT‐1 (only 43% of O3HT) and EPL‐*g*‐O3HT‐2 (65% of O3HT), may be ascribed to the better O3HT guidance by EPL backbone polymer through covalent bonding at these low‐density O3HT grafting onto EPL.

The mechanical properties of the thin film were investigated through a cyclic bending test (Figure 2E). During the test, the specimen was connected to a potentiostat to monitor current variations in real‐time. A noticeable drop in current was observed upon bending, which recovered to its original value once the film returned to its initial flat state. After ≈20 bending cycles, no significant change in current was detected, indicating that the film on the substrate maintained stable conductivity throughout the test. These results demonstrate that the EPL‐*g*‐O3HT‐1 thin film on substrate exhibits certain flexibility and mechanical resilience, highlighting its potential for use in flexible electronics.

### 2.3 Electrochemical Characterizations of EPL‐g‐O3HTs

**Figure 3:**
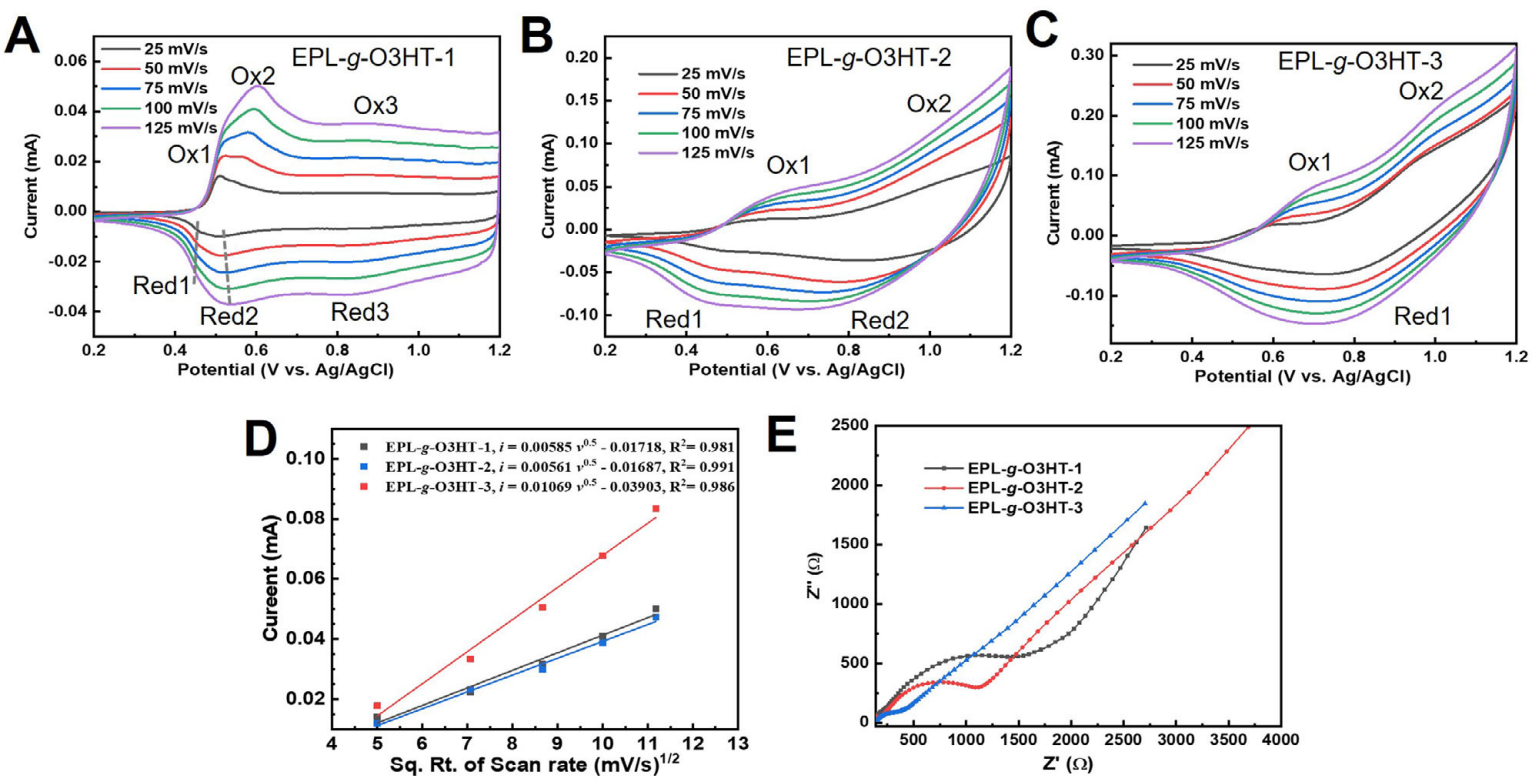
Electrochemical characterizations of EPL‐*g*‐O3HTs. Cyclic voltammograms of A) EPL‐*g*‐O3HT‐1 B) EPL‐*g*‐O3HT‐2 and C) EPL‐*g*‐O3HT‐3 on Au electrode in 0.2 M LiClO_4_ acetonitrile solution at different scan rates from 25 to 125 mV s^−1^. D) Corresponding anodic peak current versus square root of scan rate for EPL‐*g*‐O3HT‐1, EPL‐*g*‐O3HT‐2 and EPL‐*g*‐O3HT‐3. The linear fittings are R^2^>0.98. E) Electrochemical impedance spectra (EIS) of EPL‐*g*‐O3HT‐1, EPL‐*g*‐O3HT‐2 and EPL‐*g*‐O3HT‐3 in the frequency range of 0.1–100 kHz.

The electrochemical performances of EPL‐*g*‐O3HT graft copolymers with varying O3HT content were evaluated using cyclic voltammetry (CV) and electrochemical impedance spectroscopy (EIS).^[^ ^21^ ^]^ CV scans of EPL‐*g*‐O3HT‐1, EPL‐*g*‐O3HT‐2 and EPL‐*g*‐O3HT‐3 (**Figure** **3**A–C, respectively), display distinct anodic and cathodic peaks, corresponding to doping and dedoping processes. The redox peak currents increased with higher O3HT content, with EPL‐*g*‐O3HT‐1 exhibiting the lowest peak currents and EPL‐*g*‐O3HT‐3 displaying the highest. For EPL‐*g*‐O3HTs, the linear relationships between anodic peak currents and the square root of the scan rate (Figure 3D) confirm that the redox processes are diffusion‐controlled.^[^ ^5a^ ^]^

It has been reported that P3HT crystalline domains, due to the decreased bandgap, possess longer conjugation length and a smaller oxidation potential compared to disordered amorphous domains.^[^ ^22^ ^]^ In this context, Ox3/Red3 in EPL‐*g*‐O3HT‐1 and Ox2/Red2 in EPL‐*g*‐O3HT‐2 and EPL‐*g*‐O3HT‐3, can be associated with redox processes in the disordered amorphous. Ox1/Red1 in all EPL‐*g*‐O3HTs can be assigned to the redox reactions occurring at the crystalline domains of O3HT within them. Due to the proximity of Ox2 (0.60 – 0.65 V) in EPL‐*g*‐O3HT‐1 and the Ox1 (0.60 – 0.70 V) in EPL‐*g*‐O3HT‐2 and EPL‐*g*‐O3HT‐3, Ox2/Red2 in EPL‐*g*‐O3HT‐1 can also be related to the crystalline domains of O3HT. The appearance of two crystalline domains‐related peaks (Ox1 and Ox2) in EPL‐*g*‐O3HT‐1 may result from two distinct crystalline domains with slightly different degrees of order introduced by the grafted side chains.^[^ ^23^ ^]^ In addition, EPL‐*g*‐O3HT‐1 exhibits the lowest oxidation peak potentials of crystalline domains (≈0.50 V), while EPL‐*g*‐O3HT‐3 shows the highest (0.65 – 0.70 V). That indicates that O3HT crystalline domains within EPL‐*g*‐O3HT‐1 have a longer conjugation length compared to those in EPL‐*g*‐O3HT‐2 and EPL‐*g*‐O3HT‐3, consistent with the findings from Raman spectroscopy.

EIS was employed to investigate the charge transfer in EPL‐*g*‐O3HTs (Figure 3E). A decrease in charge transfer resistance (R_ct_) of EPL‐*g*‐O3HTs is observed as the O3HT content increases. This reduction in R_ct_ demonstrates enhanced electroactivity and faster charge transfer kinetics in the films with higher grafting densities of EPL‐*g*‐O3HTs. The EIS results are consistent with the CV measurements, where EPL‐*g*‐O3HT‐3 (90 wt.% O3HT) shows the highest redox peak currents (Figure 3C). Conductivity measurements further validate these findings (Table S3, Supporting Information). The conductivity of EPL‐*g*‐O3HT‐1 was 1.13 ± 0.03 S m^−1^, of EPL‐*g*‐O3HT‐2 1.36 ± 0.02 S m^−1^ and of EPL‐*g*‐O3HT‐3 1.98 ± 0.05 S m^−1^. Additionally, the conductivity of EPL‐*g*‐O3HT‐1 exhibited clear temperature dependence. Increasing the temperature from 25 to 40 °C resulted in a threefold increase in conductivity (Figure S6, Supporting Information).^[^ ^24^ ^]^ The electronic conductivity of EPL‐*g*‐O3HT‐1, as measured by the four‐point probe method, exhibited stability across varying relative humidity (RH) environments, demonstrating humidity‐independent behavior. Increasing the temperature from 25 to 40 °C resulted in a threefold increase in conductivity (Figure S6, Supporting Information). The electronic conductivity of EPL‐*g*‐O3HT‐1, as measured by the four‐point probe method, exhibited stability across varying relative humidity (RH) environments, demonstrating its humidity‐independent electronic behavior. This observed humidity independence aligns with the previous report (Figure S7, Supporting Information).^[^ ^25^ ^]^ Notably, the same three thin‐film samples were subjected to repeated measurements over a five‐day period under fluctuating RH conditions, further corroborating the robust conductivity stability of EPL‐*g*‐O3HT‐1 under environmental stress. The good conductivity performance of EPL‐*g*‐O3HTs highlights their potential in thin‐film polymer electronics devices.

### 2.4 Degradation of EPL‐g‐O3HTs Thin Films

**Figure 4:**
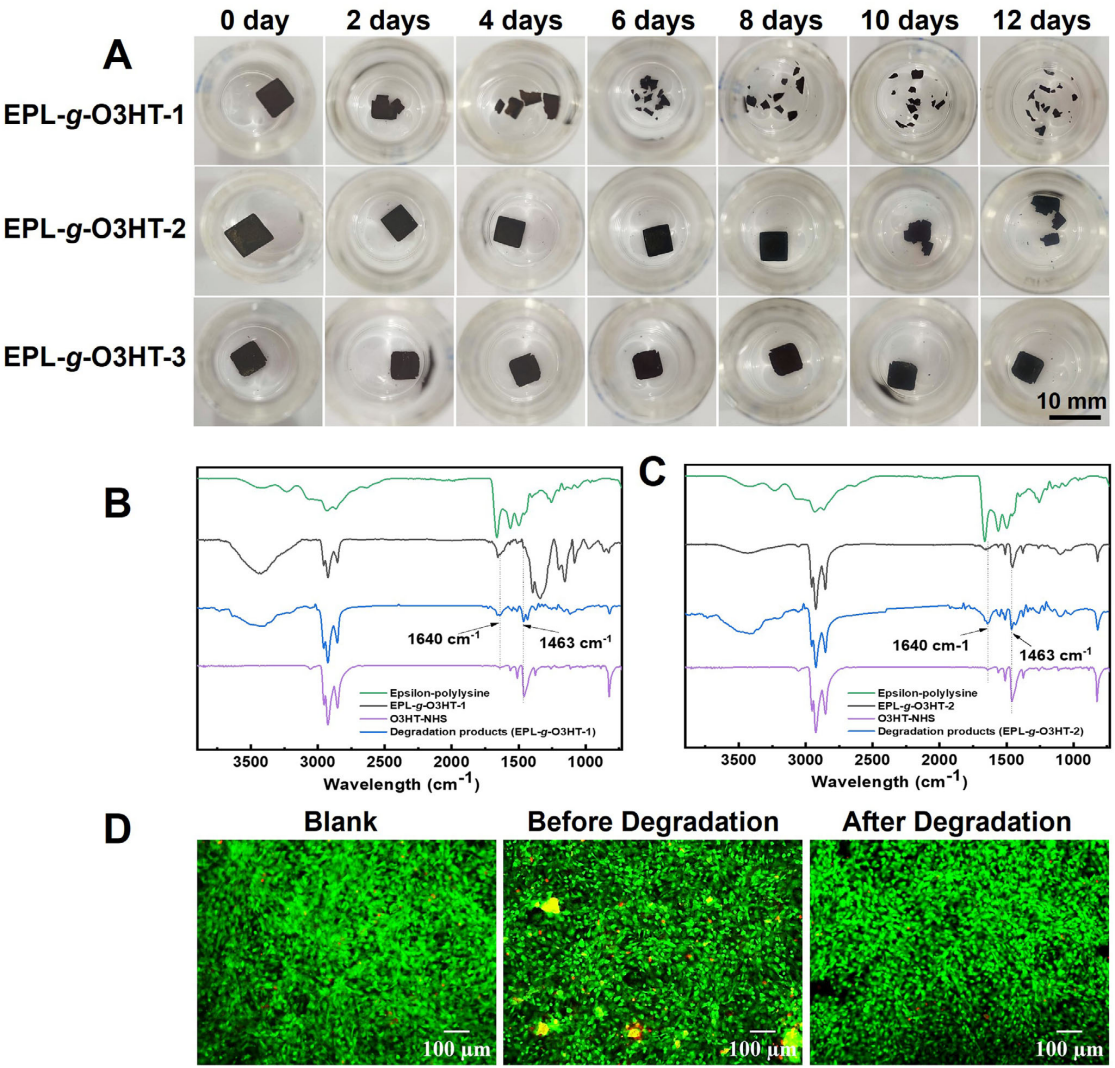
Enzymatic degradation of EPL‐*g*‐O3HTs thin films. A) Optical images of EPL‐*g*‐O3HTs thin films over a 12‐days of enzymatic degradation in PBS. B) and C) FTIR spectra of EPL‐*g*‐O3HT‐1 and EPL‐*g*‐O3HT‐2 thin films before and after enzymatic degradation, compared with pristine O3HT‐NHS and ɛ‐poly‐L‐lysine. D) In vitro biocompatibility of EPL‐*g*‐O3HT‐1 before and after degradation measured by LIVE/DEAD staining assay.

Understanding the degradation behavior of transient electronic materials is essential for developing devices that safely disintegrate after use, thereby minimizing electronic waste and reducing environmental impact.^[^ ^3^, ^26^ ^]^ To this end, evaluating the degradation performance of EPL‐*g*‐O3HTs copolymers is critical in assessing their viability in transient polymer electronics. The backbone polymer in EPL‐*g*‐O3HTs, ε‐poly‐L‐lysine (EPL), primarily degrades through enzymatic cleavage of its peptide bonds by specific enzymes, such as trypsin and proteinase K.^[^ ^27^ ^]^ Therefore, we hypothesized that EPL‐*g*‐O3HTs should exhibit regulated degradation performance depending on the grafting density, i.e., the content of O3HT. We conducted an enzymatic degradation experiment on EPL‐*g*‐O3HTs thin films, using trypsin as the enzymatic agent. A water‐soluble dextran layer served as the sacrificed layer during the thin film preparation (Figure S8, Supporting Information).^[^ ^15b^ ^]^ Over 12 days under enzymatic conditions in PBS, the degradation profiles of EPL‐*g*‐O3HT‐1, EPL‐*g*‐O3HT‐2 and EPL‐*g*‐O3HT‐3 films displayed distinct behaviors (**Figure** **4**A), while O3HT‐NHS thin film remained stable with no observed degradation (Figure S9, Supporting Information). By day 2, EPL‐*g*‐O3HT‐1 showed early signs of degradation, including visible cracks and fragmentation, which broke down into tiny pieces by day 12 (end of the degradation study). The rapid degradation of EPL‐*g*‐O3HT‐1 is due to its high ε‐poly‐L‐lysine content (57 wt.%). In contrast, EPL‐*g*‐O3HT‐2 and EPL‐*g*‐O3HT‐3 exhibited slower degradation, remaining largely intact without observed structural changes during the early stages. EPL‐*g*‐O3HT‐2 began showing visible cracks by day 10 and fragmented into large sections by day 12, while EPL‐*g*‐O3HT‐3 displayed the greatest resistance to enzymatic degradation, exhibiting only minor cracks by day 12 (Figure S10, Supporting Information). Its high resistance to enzymatic degradation can be attributed to its higher O3HT and lower EPL content.

Further analysis of EPL‐*g*‐O3HT‐1 and EPL‐*g*‐O3HT‐2 thin films, before and after degradation, was conducted using FTIR spectroscopy. The FTIR spectra of degradation products (Figure 4B,C) revealed increased peak intensity at 1640 cm^−1^, associated with C ═ O stretching within the O3HT‐NHS component. This increase corresponds to a higher relative content of O3HT in the degradation products, as sections of ɛ‐poly‐L‐lysine were cleaved from the copolymer during the enzymatic degradation. Additionally, a new peak at 1463 cm^−1^ appeared, corresponding to the C‐H bending of hexyl chains in O3HT,^[^ ^28^ ^]^ which can also be attributed to the increased relative content of O3HT within the degradation components. The FTIR results confirm structural change within the copolymer during enzymatic degradation. These findings demonstrate the rapid, controlled degradation observed in EPL‐*g*‐O3HT‐1, suggesting its strong potential for applications in transient electronics where predictable disintegration is essential. EPL‐*g*‐O3HT‐2 (and EPL‐*g*‐O3HT‐3 to a smaller extent) also underwent enzymatic degradation, albeit much slower. Table S4 (Supporting Information) provides a comparison table benchmarking the key parameters of EPL‐*g*‐O3HT‐1 against other conducting polymer‐based biodegradable electronic materials from the literature. Therefore, EPL‐*g*‐O3HT‐1 was selected as the graft copolymer for subsequent investigations.

The biocompatibility of EPL‐*g*‐O3HT‐1 before and after degradation was evaluated using LIVE/DEAD staining assay. As shown in Figure 4D, both the original and degraded EPL‐*g*‐O3HT‐1 samples exhibited cell viability comparable to the blank control. Strong green fluorescence from calcein‐AM indicated viable cells, while red fluorescence from EthD‐1 that marks dead cells, was negligible. These results suggest that EPL‐*g*‐O3HT‐1 maintains good biocompatibility before and after degradation.

### 2.5 Antibacterial Performance of EPL‐g‐O3HT‐1

Antibacterial properties are important for wearable electronics materials as these devices remain in constant contact with skin and are exposed to sweat, moisture and bacteria, which can lead to skin irritation or infections over time.^[^ ^6^, ^29^ ^]^ Incorporating antibacterial features into wearable electronics made by polymeric materials can effectively suppress bacterial proliferation and ensure hygiene at the skin‐contact interface.^[^ ^29b^ ^]^ ε‐Poly‐L‐lysine (EPL) is well‐known for its broad‐spectrum bactericidal properties, with many studies reporting the antibacterial capability of EPL‐based materials.^[^ ^8^, ^30^ ^]^ Inspired by this, the antibacterial activity of biodegradable EPL‐*g*‐O3HT‐1 electronic material was investigated against both Gram‐positive *S. aureus* and Gram‐negative *E. coli*.

**Figure 5:**
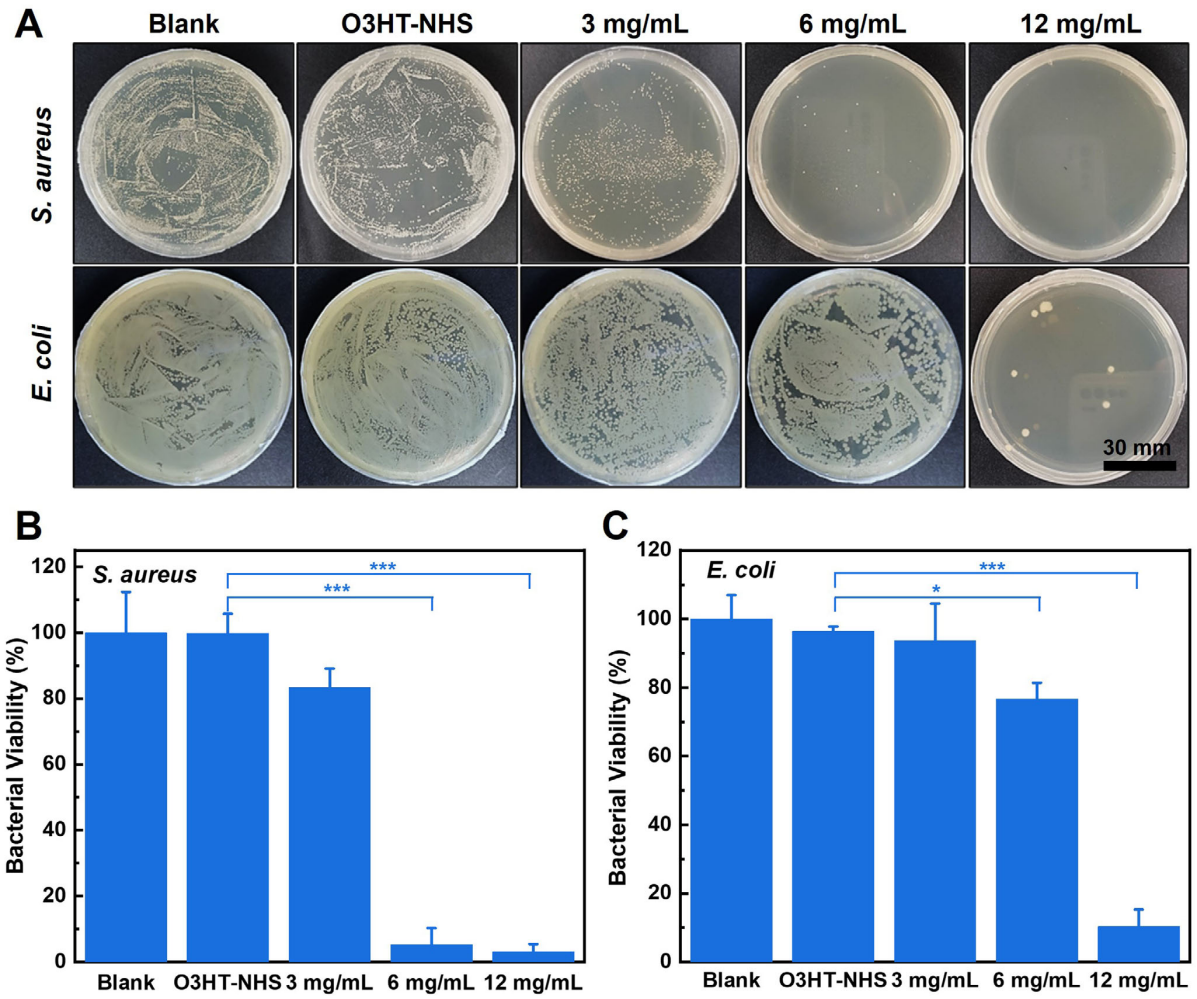
Antibacterial performance of EPL‐*g*‐O3HTs copolymers. A) Photographs of the survival bacterial colonies on agar plates to demonstrate in vitro antibacterial activities of O3HT‐NHS and EPL‐*g*‐O3HTs copolymers against *E. coli* and *S. aureus*, respectively. B) The bacterial viability of O3HT‐NHS and EPL‐*g*‐O3HTs copolymers against *E. coli* and *S. aureus*, respectively. Data presented as mean ± SD, *n* = 3, and P‐values are calculated using an unpaired t‐test performed in GraphPad Prism 8. *p<0.05, ***p<0.001.

When EPL‐*g*‐O3HT‐1 copolymer was co‐cultured with bacterial suspensions for 24 h, a notable reduction in the survival rate of both *S. aureus* and *E. coli* on agar plates was observed at the concentration of 6 mg mL^−1^, with a more significant reduction observed at 12 mg mL^−1^, compared to the untreated control group (**Figure** **5**A). In contrast, the group treated with O3HT‐NHS, which lacks EPL, showed no antibacterial effects, displaying results similar to the negative control (Figure S11, Supporting Information). Quantitative analysis further reveals that the treatment with the highest concentration (12 mg mL^−1^) of EPL‐*g*‐O3HT‐1 investigated achieved a bactericidal efficacy of 89.6% against *E. coli* and 97% against *S. aureus* (Figure 5B,C). It is noteworthy that even at 6 mg mL^−1^, EPL‐*g*‐O3HT‐1 demonstrated a remarkable 94.6% bactericidal efficacy against *S. aureus*. This trend of Gram‐positive *S. aureus* being more sensitive to EPL than Gram‐negative *E.coli* agrees with previous reports,^[^ ^31^ ^]^ and might be related to the different cell membrane structures and constituents between Gram‐positive and Gram‐negative bacteria. The excellent antibacterial performance of the EPL has been attributed to disruption of the structural integrity of bacterial membranes, causing the release of some cellular components and ultimately leading to cell death.^[^ ^31^, ^32^ ^]^ It is worth noting that the antibacterial tests in this study were conducted using a powder form of EPL‐*g*‐O3HT to allow for systematic and concentration‐dependent evaluation. The results of antibacterial tests demonstrate the antibacterial properties of transient polymer electronics material EPL‐*g*‐O3HT‐1.

### 2.6 Applications Demonstration of EPL‐g‐O3HT‐1

**Figure 6:**
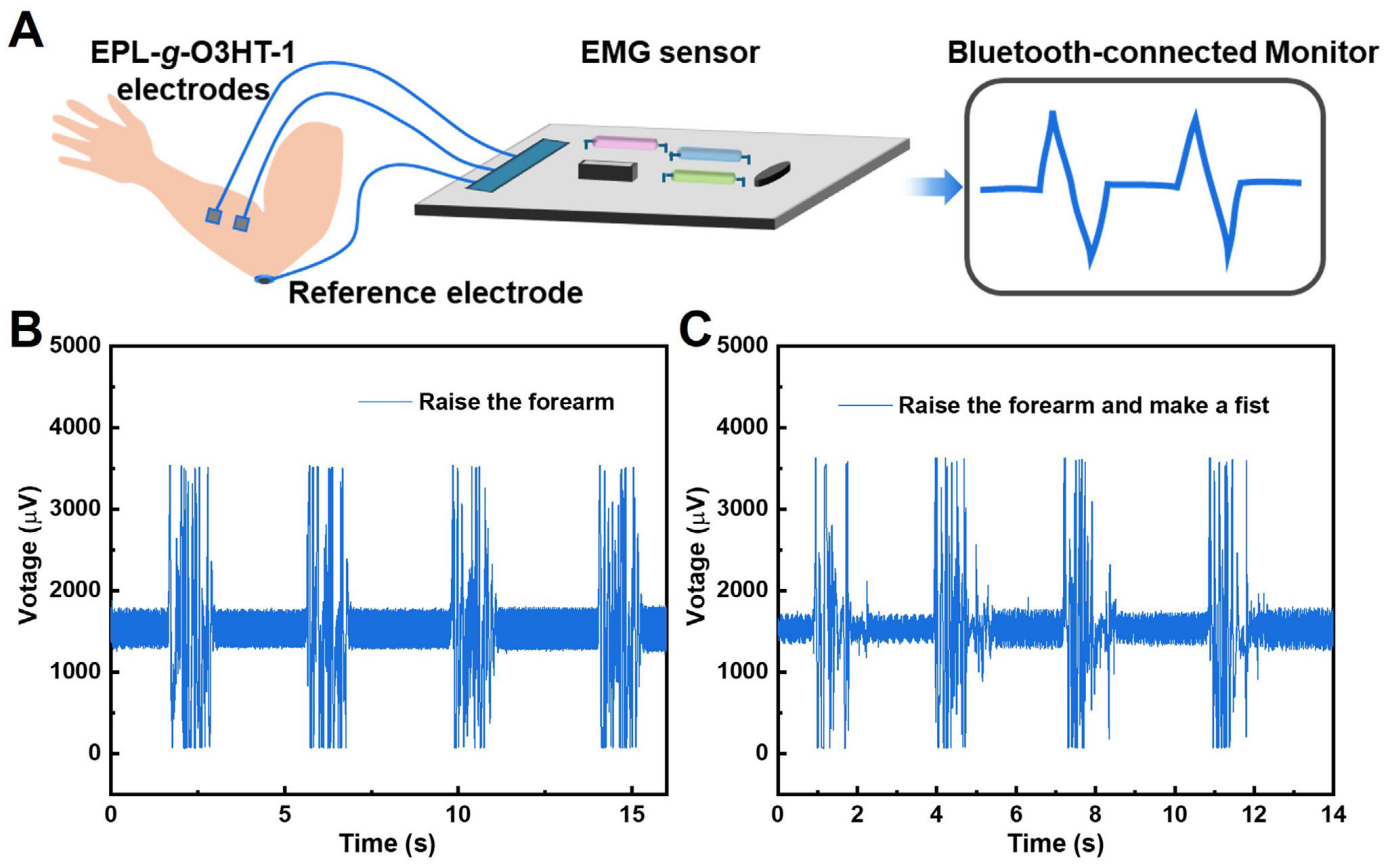
Demonstration of an application of EPL‐*g*‐O3HT‐1 as electromyogram (EMG) sensors. A) Schematic setup for the EMG measurement using the EPL‐*g*‐O3HT‐1 copolymer. EMG signals from the forearm muscle were recorded during repeated forearm‐raising motions, with B) or without C) fist‐clenching. The peaks in the signals are caused by muscle contractions.

As EPL‐*g*‐O3HT‐1 manifested considerable conductivity, enzymatic degradability and broad‐spectrum antibacterial properties, it was considered that it holds excellent potential for use in transient wearable electronics.^[^ ^6^, ^33^ ^]^ The schematic representation and the photograph of an EPL‐*g*‐O3HT‐1‐based transient electromyography (EMG) sensor is shown in **Figures** **6**A and S12 (Supporting Information), respectively. Two EPL‐*g*‐O3HT‐1 thin films were attached to the same muscle of the volunteer's forearm, working as active electrodes to collect EMG signals.^[^ ^34^ ^]^ A third electrode was placed on the elbow (a non‐active muscle) as the ground electrode for referencing the electrical signals.^[^ ^34^ ^]^ The sensor performance was evaluated during different forearm motions. Figure 6B presents the real‐time EMG signal generated as the volunteer raised their forearm. Figure 6C shows the sensing response to the volunteer raising their forearm and clenching the fist at the end. In addition to the similar response during the forearm‐raising period, an extra signal was detected at the end of each repeated motion cycle, corresponding to the fist‐clenching action. Additional data in Figure S13 (Supporting Information) presents the EMG signal recorded as the volunteer slid their arm outward along a desk, keeping the angle between the arms constant. The signal response of this motion was markedly different to the signal response generated by forearm‐raising motion, with or without the fist‐clenching. The corresponding EMG signals, which indicate muscle contraction strength and are obtained after filtering and processing the raw EMG data, exhibit similar trends (Figure S14, Supporting Information). These variations in the EMG signal demonstrate that the EPL‐*g*‐O3HT‐1‐based EMG sensor is sensitive to detect and differentiate various muscle activities, illustrating its great potential for application in transient wearable electronics.

**Figure 7:**
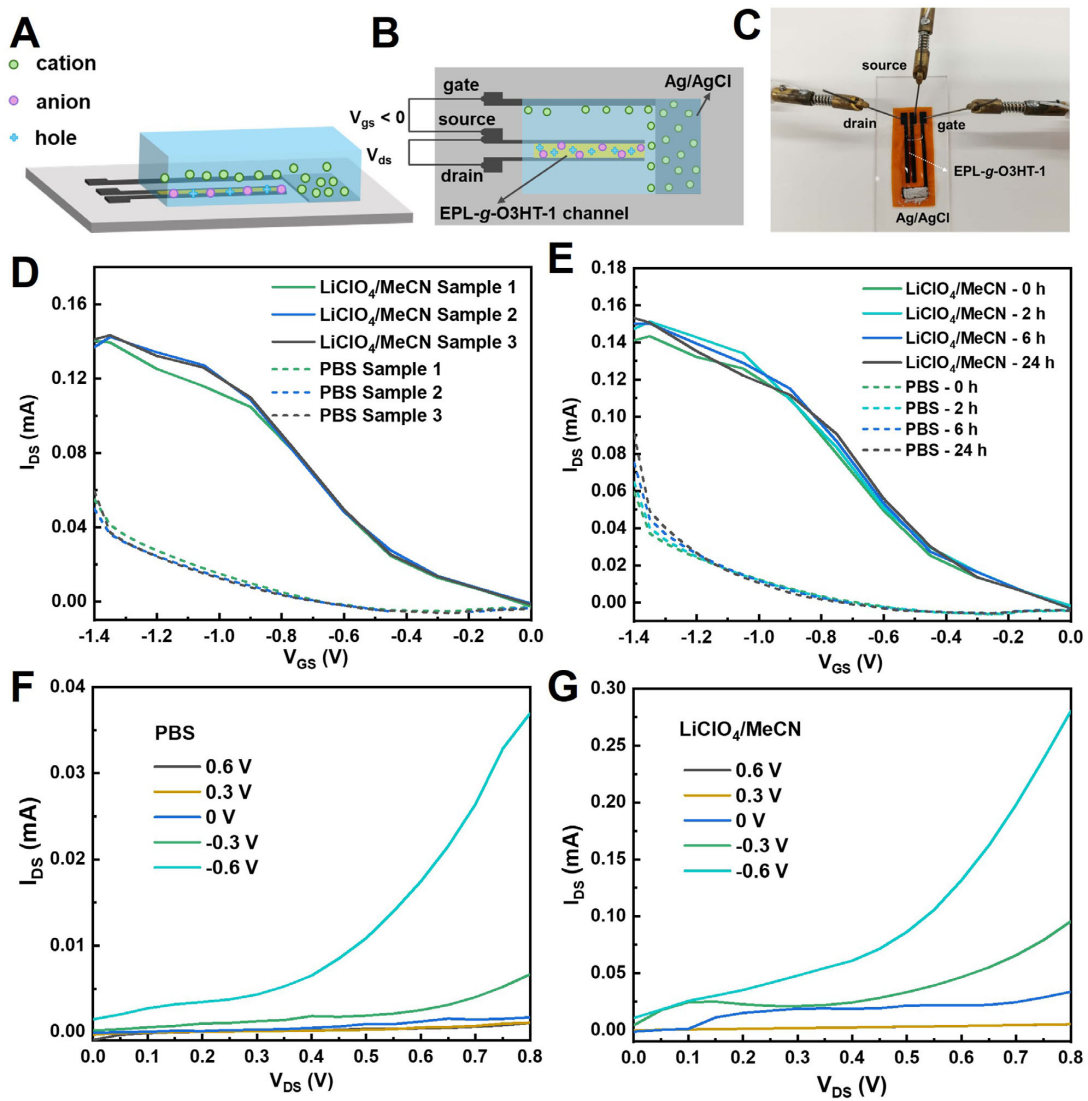
Demonstration of an application of EPL‐*g*‐O3HT‐1 as OECT devices. Device structure of a typical OECT is shown from the side perspective A) and the top perspective B) in its “on” state. C) Photograph of the fabricated OECTs. D) Transfer characteristics of three EPL‐*g*‐O3HT‐1 based OECTs under different gate voltages in PBS buffer and in LiClO_4_/acetonitrile as the electrolytes. E) Transfer characteristics of Sample 3 under different gate voltages in PBS buffer and in LiClO_4_/acetonitrile as the electrolytes. Output curves displaying the drain current versus drain voltage under varying gate voltages for the OECT in PBS buffer F) and LiClO_4_/acetonitrile (G) as the electrolyte, respectively.

O3HT, as a known p‐type conducting polymer, can undergo unique doping or dedoping in response to different potentials.^[^ ^33^, ^35^ ^]^ This property also makes EPL‐*g*‐O3HT‐1 an excellent candidate for p‐type organic electrochemical transistors (OECTs), extending its versatility beyond wearable sensors. To investigate the feasibility of utilizing EPL‐*g*‐O3HT‐1 for transistor‐based applications, we constructed a transistor using EPL‐*g*‐O3HT‐1 as the channel material connecting the source and drain electrodes. The device employed an Ag/AgCl electrode (versus 3 M NaCl) as the electrolyte‐gated electrode and laser‐scribed graphene (LSG) as the source and drain electrodes (**Figure** **7**A–C). PBS buffer and LiClO_4_ in acetonitrile were used as electrolytes to evaluate the transistor's transfer and output characteristics, respectively. The “ON/OFF” state of the EPL‐*g*‐O3HT‐1 channel was controlled by applying different potentials to the gate electrode via the electrolyte. The transfer characteristics of the three prepared OECTs in different electrolytes are shown in Figure 7D, demonstrating negligible sample‐to‐sample variation. The 24 h operational stability was assessed by comparing the transfer characteristics of sample 3 over time (Figure 7E), which remained nearly stable throughout the measurement period. Output characteristics of EPL‐*g*‐O3HT‐1‐based OECT were shown in Figure 7F,G. When a negative voltage was applied to the gate electrode, anions from the electrolyte were injected into the EPL‐*g*‐O3HT‐1 channel, tuning the electronic charge density, leading to doping of EPL‐*g*‐O3HT‐1 and the increase in the conductivity of the channel.^[^ ^33^, ^35^, ^36^ ^]^ In contrast, applying a positive voltage caused cations from the electrolyte to diffuse into the channel, leading to the dedoping of EPL‐*g*‐O3HT‐1 and turning the transistor off.^[^ ^33^, ^35^, ^36^ ^]^ These results demonstrate that OECTs based on EPL‐*g*‐O3HT‐1 exhibit characteristic p‐type transistor behavior. The related transconductance (*g*_m_)of sample 3 at 0 h was estimated by the first derivative of the transfer characteristics (Figure S15, Supporting Information). When using acetonitrile containing LiClO_4_ as electrolyte, the transconductance value (0.20 mS) is higher than that of PBS (0.10 mS). The on‐to‐off current ratio using acetonitrile containing LiClO_4_ is ≈0.3, which is similar to our recently reported value of 0.6 for the similar degradable graft copolymer system poly(caprolactone‐co‐3‐alkyne‐valerolactone)‐graft‐oligo‐3‐hexylthiophene.^[^ ^15b^ ^]^ When output characteristics of EPL‐*g*‐O3HT‐1‐based OECT devices in different electrolytes, a higher on‐to‐off current ratio was obtained in acetonitrile containing LiClO_4_, which could be ascribed to the superior doping behavior of the slightly hydrated ClO_4_^−^ ions (compared to highly hydrated Cl^−^ ions in PBS),^[^ ^22^, ^37^ ^]^ enabling faster and stable doping‐dedoping transitions in the hydrophobic O3HT within EPL‐*g*‐O3HT‐1 channel.^[^ ^22^, ^37^ ^]^ To further enhance high signal amplification during ion‐to‐electron transduction, the channel geometry of the EPL‐*g*‐O3HT‐1‐based OECT was optimized to improve steady–state performance (i.e., transconductance (*g*_m_)). The transfer characteristic and the corresponding transconductance of the best‐performing device are presented in Figure S16B (Supporting Information). An improved channel current in the accumulation mode OECTs was achieved, resulting in lower operating voltage by the device configuration with gold source and drain (S/D) electrodes. A high transconductance value of 5.6 mS was calculated for this OECT. For comparison, Nightingale et al. previously reported a maximum transconductance of 4 mS for P3HT‐based OECTs.^[^ ^38^ ^]^ A clear color change was also observed during device operation, indicating electrochemical doping of the polymer (Figure S16A, Supporting Information). The results highlight the great potential of EPL‐*g*‐O3HT‐1‐based OECTs for signal amplification in electrochemical transistors, particularly for integration into wearable health monitoring devices.

## 3 Conclusions

In this work, we designed and synthesized graft copolymers of O3HT and ɛ‐poly‐L‐lysine, EPL‐*g*‐O3HTs. ɛ‐Poly‐L‐lysine served as a biodegradable polymer backbone, covalently grafted with O3HT chains in three different grafting densities (43, 65 and 90 wt.% relative to ɛ‐poly‐L‐lysine). This new class of copolymers address key challenges in transient electronics by integrating significant conductivity, controlled enzymatic degradability (over 12 days) and potent antimicrobial properties (achieving ≈90% bactericidal efficacy against *E. coli* and *S. aureus* at a 12 mg mL^−1^ concentration) into one macromolecule. The synergistic combination of these properties enabled the successful demonstration of EPL‐*g*‐O3HTs as EMG sensors and OECTs, highlighting its versatility and suitability for biocompatible transient electronic devices. This innovative strategy of engineering multiple functionalities within a single graft copolymer offers a promising pathway for advancing sustainable and biodegradable electronic technologies tailored to desired properties for diverse applications.

## 4 Experimental Section

### Synthesis of ɛ‐poly‐L‐lysine‐*graft*‐oligo(3‐hexylthiophene)

Oligo(3‐hexylthiophene) end‐capped with *N*‐hydroxysuccinimide esters (O3HT‐NHS) was synthesized utilizing our previous reported method to first obtain O3HT end‐capped with azide^[^ ^15b^ ^]^ and further modified with NHS end‐capped groups (O3HT‐NHS) (Schemes S1 and S2 and Figure S17, Supporting Information). Detailed procedures were described in the Supporting Information, Section 2.

ε‐Poly‐L‐lysine‐*graft*‐oligo(3‐hexylthiophenes) (EPL‐*g*‐O3HTs) were synthesized by conjugating O3HT‐NHS onto ɛ‐poly‐L‐lysine through the peptide bonds formation between amine of ε‐poly‐L‐lysine and NHS of O3HT‐NHS. O3HT‐NHS (50 mg, ≈0.011 mmol) was dispersed in a mixture of THF (20 mL) and DMSO (10 mL). Separately, a solution of ε‐poly‐L‐lysine (500 mg, ≈0.100 mmol) in deionized water (18.2 MΩ cm, 10 mL) was prepared. The pH of the ε‐poly‐L‐lysine solution was adjusted to 8 using 1 M NaOH to facilitate the reaction. Then, the ε‐poly‐L‐lysine solution was added to the O3HT‐NHS solution, and the resulting mixture was stirred at room temperature in the dark for 24 h. Following this, the reaction solution was precipitated in cold acetone for ≈15 min and washed three times with water (50 mL × 3) to remove unreacted water‐soluble ε‐poly‐L‐lysine. The collected filtrate was dissolved in DCM and gradually added to ice‐cold acetone again. A fractional precipitation process was then performed to remove unreacted O3HT‐NHS and purify the EPL‐*g*‐O3HT copolymer. The mixture was allowed to stand for 5 min and the precipitation was collected by filtration. The resulting precipitates were extracted with CHCl_3_ and condensed by a rotary evaporator. The purified EPL‐*g*‐O3HT was dried in a vacuum oven for 12 h before storage. The EPL‐*g*‐O3HT copolymer obtained using this procedure was labelled as EPL‐*g*‐O3HT‐1. EPL‐*g*‐O3HT‐2 and EPL‐*g*‐O3HT‐3 were synthesized using the same procedure, only with increased O3HT‐NHS amounts. The feed ratio of O3HT‐NHS: ε‐poly‐L‐lysine was 100 mg: 500 mg for EPL‐*g*‐O3HT‐2 and 250 mg: 500 mg for EPL‐*g*‐O3HT‐3.

### Characterization of EPL‐*g*‐O3HTs

The ^1^H NMR spectra were recorded using a Bruker Avance III 400 MHz spectrometer. Solvents for NMR, specifically CDCl_3_​ and D_2_​O, were sourced from Sigma–Aldrich and utilized as provided. Chemical shifts (δ) are reported relative to tetramethylsilane (TMS) and deuterochloroform (CDCl_3_), with values expressed in parts per million (ppm).

FTIR spectra were recorded in transmission mode using a Perkin Elmer FTIR spectrometer (Massachusetts, USA), collecting over 16 scans, covering a wavenumber range of 400 to 4000 cm^−1^.

Elemental analysis was conducted using the Elemental Analyzer (vario EL cube, Elementar Analysensysteme GmbH), with ≈2 mg of each sample.

GPC was employed to determine the molecular weights of the polymers, utilizing a Shimadzu Nexera GPC system equipped with a Refractive Index detector (RID)‐20A. Polymer solutions were prepared by dissolving the samples in THF at a 5 mg mL^−1^ concentration, followed by filtration through 0.22 µm PTFE syringe filters before injection into the GPC system. Detailed operational parameters of the GPC system can be found in Supporting Information. The synthesized O3HT‐NHS had a number‐average molecular weight (*M*_n_) of 4411 Da and a weight‐average molecular weight (*M*_w_) of 5813 Da, resulting in a dispersity (Đ) of 1.32 (Figure S18, Supporting Information).

UV‐Vis spectra were measured on a Shimadzu UV‐Visible 2600 spectrophotometer with 3.5 mL quartz cuvettes. The absorbance (Abs.) of O3HT in THF solutions was recorded across varying concentrations of O3HT, and the data were plotted to generate a calibration curve: *Abs*. = 0.00364 *Con*.(O3HT) – 0.01567, with an R^2^ value of 0.996. The calibration curve was employed to estimate the weight of O3HT in the graft copolymers by multiplying the concentration of O3HT (µg mL^−1^) by the solution volume (mL). The weight percentage of O3HT in EPL‐*g*‐O3HT was then calculated according to the equation: wt.% (O3HT) = [(weight of O3HT in EPL‐*g*‐O3HT) / (weight of EPL‐*g*‐O3HT)] × 100%.

DSC was conducted on a Q1000 TA instrument under a 50 mL min^−1^ nitrogen flow rate. The analysis involved a heat/cool/heat cycle sequence: initial heating from ambient temperature to 150 °C at a rate of 20 °C min^−1^, followed by cooling to −80 °C at 10 °C min^−1^, and a final heating phase from −80 to 230 °C at 20 °C min^−1^. Each sample weighed ≈8 to 10 mg.

TGA was performed using TA Instruments TGA Q5000. Samples, each weighing between 5 and 10 mg, were heated from 35 to 700 °C at a rate of 20 °C min^−1^ under a nitrogen atmosphere with a purge flow rate of 50 mL min^−1^.

Cyclic voltammetry (CV) experiments were conducted on a CH Instruments (USA) electrochemical workstation (CHI660E). The setup utilized a standard three‐electrode configuration comprising a gold working electrode (1.6 mm in diameter), an Ag/AgCl reference electrode, and a platinum wire as the counter electrode. Polymer solutions (15 µL, 5 mg mL^−1^) were drop‐cast onto the gold electrode and dried under reduced pressure for 30 min. Further experimental details are provided in Supporting Information.

Conductivity measurements were performed using the four‐point probe (Jandel RM3000+) technique, with current applied through the outer probes and the voltage measured between the inner probes to minimize contact resistance. Thin films of EPL‐*g*‐O3HT‐1, EPL‐*g*‐O3HT‐2, and EPL‐*g*‐O3HT‐3 were prepared by drop‐casting 100 µL of 40 mg mL^−1^ sample solutions in THF onto pre‐cut glass slides (10 mm × 10 mm) that had been cleaned using UV‐ozone (Jelight UVO cleaner, model 342‐220). After deposition, the films were dried under ambient conditions to ensure uniform distribution across the substrate. The films were then doped by immersion in 1 M FeCl_3_ in acetonitrile for 30 s, followed by two washes with fresh acetonitrile to remove excess dopant. After doping, the films were dried overnight in a desiccator. The thickness was obtained using a height gauge (Mitutoyo Model ID‐H0530E). Humidity environments with different relative humidity (RH) levels were established using saturated salt solutions: MgCl_2_ for 33% RH, Mg(NO_3_)_2_ for 52% RH, NaCl for 75% RH, and KCl for 86% RH.^[^ ^40^ ^]^ Samples were placed in the humidity chambers 24 h prior to conductivity measurements.

*I*‐*V* curves of EPL‐*g*‐O3HT‐1 under various temperatures on the hot plates were obtained using a Palmsens 4 potentiostat with a two‐electrode setup. The EPL‐*g*‐O3HT‐1 solution in THF (40 mg mL^−1^, 100 µL) was drop‐cast onto the PET substrate (8 mm × 10 mm) and dried under reduced pressure for 4 h. The films were then doped by immersion in 1 M FeCl_3_ in acetonitrile for 30 s, followed by two rinses with fresh acetonitrile to remove excess dopant. Samples were then dried overnight in a desiccator. The length of the sample (5 mm) was the distance between the two clamps at which the films were attached. *I*–*V* curves were recorded by sweeping the potential from 0 to 3 V and measuring the resulting current. Electrical conductivity was calculated based on Pouillet's law.

Bending test samples were prepared by drop‐casting EPL‐*g*‐O3HT‐1 solution (40 mg mL^−1^, 150 µL) onto PET substrates (8 mm × 20 mm), followed by doping and drying procedures identical to those used for other thin film preparations. Real‐time conductivity changes during mechanical deformation were also monitored by performing *I*‐t measurements using a two‐electrode setup connected to a potentiostat.

Surface topography was characterized using an MFP‐3D Origin AFM (Oxford Instruments, Asylum Research) in tapping mode, with silicon probes operating at a nominal resonance frequency of 150 kHz (Budget Sensors, USA). Thin film samples were prepared by spin‐coating 15 µL of 5 mg mL^−1^ polymer solutions in THF onto freshly cleaved mica. The films were doped following the protocol as described above. After doping, the samples were annealed at 80 °C for 120 min and then cooled gradually to 35 °C at a rate of 1 °C per minute.

Raman spectroscopy measurements were conducted using a LabRAM HR Evolution confocal Raman Microscope (Horiba, Japan) with a 785 nm laser (100 mW) as the light source. The laser passed through a 99% neutral density filter and was focused on the sample using a 10× objective lens (Olympus, N.A. 0.55). The spectra were collected at room temperature in the wavenumber range of 600–1800 cm^−1^. Each measurement was taken over 60 s with 5 repeated scans, using a confocal hole size of 100 or 200 µm and a 600 l mm^−1^ diffraction grating.

X‐ray diffraction (XRD) patterns were collected using a PANalytical Empyrean diffractometer with Cu Kα radiation (λ = 0.15406 nm, 8.04 keV) at The University of Auckland's Micro characterization Facility. Data acquisition was performed over a scattering angle of 3.5–50° (2θ), with each scan taking ≈45 min. A beam knife was employed to reduce stray X‐rays. XRD samples were prepared by drop‐casting 20 µL of a 20 mg mL^−1^ solution onto Si (100) wafers, which were air‐dried for 10 min. The spectra were normalized against a blank Si wafer as a reference. Doping and annealing followed the procedure described in the AFM characterization. The d‐spacing values were calculated according to the equation *d* = *λ*/2sin*θ*, where *λ* is 1.5406 Å, *θ* is half of the 2*θ* angle. The crystallite sizes L were calculated using the Scherrer equation: L = 0.9 *λ*/(*β* × cos *θ)*, where λ represents the wavelength of the incident x‐rays, *β* is the full width at half maximum (FWHM) of diffraction peak in radians, and *θ* is the Bragg angle.^[^ ^20^ ^]^

### Enzymatic Degradation

The enzymatic degradation of O3HT‐NHS and EPL‐*g*‐O3HTs thin films was investigated using trypsin at a concentration of 1 mg mL^−1^ in PBS, maintained at 37 °C. A sacrificial dextran layer was incorporated during the thin film preparation process to facilitate the formation of free‐standing thin films. Prior to casting the sample solutions (40 mg mL^−1^, 100 µL), a 100 µL dextran solution (5% w/v) was drop‐cast onto PET substrates, pre‐cut to 10 mm × 10 mm, and allowed to dry under ambient conditions. After the sample solutions had been drop‐cast and dried, the PET substrates were immersed in PBS at 60 °C. After ≈4 to 6 h, free‐standing polymer thin films were observed floating on the surface. At this stage, the PET substrates were carefully removed. Once the PBS was cooled to ≈37 °C, trypsin was added to achieve a final concentration of 1 mg mL^−1^. Optical images were captured at specified intervals to monitor and analyze the degradation dynamics of O3HT‐NHS and EPL‐*g*‐O3HTs thin films. Transmission FTIR was employed to analyze the structure of degradation fragments of EPL‐*g*‐O3HTs.

The in vitro cytotoxicity of EPL‐*g*‐O3HT‐1 before and after degradation was evaluated using LIVE/DEAD cell staining. EPL‐*g*‐O3HT‐1 and degradation products (obtained through enzymatic degradation as previously described) were dissolved in THF at a concentration of 5 mg mL^−1^ and spin‐coated onto glass slides (10 × 10 mm). Blank glass slides served as controls. Following UV sterilization, mouse NIH/3T3 fibroblasts were seeded into 24‐well plates containing the sample‐coated slides. Custom‐made PDMS sample holders were put into the well to restrict the cell growth area to 5 × 5 mm. FF‐20 medium was used for cell culture. After 48 h, the medium was removed, and the cells were washed three times with F‐10 medium. Then, 100 µL of LIVE/DEAD staining solution was applied, and the samples were incubated at room temperature for 30 min prior to fluorescence imaging using a Nikon ECLIPSE Ni‐U microscope.

### Antibacterial Test

The antibacterial properties of EPL‐*g*‐O3HT‐1 were investigated in vitro against Gram‐negative *Escherichia coli* (*E. coli*) and Gram‐positive *Staphylococcus aureus* (*S. aureus*). For these tests, 1.5, 3, and 6 mg of UV‐sterilized EPL‐*g*‐O3HT‐1, along with 6 mg of O3HT‐NHS as a reference material, were immersed in 0.5 mL of a bacterial suspension (10⁶ CFU mL^−1^) and incubated at 37 °C for 24 h. Streptomycin was employed as the positive control, while the blank control group remained untreated. After incubation, bacterial suspensions were collected, and their optical density at 600 nm (OD600) was measured to assess bacterial viability using a Multimode plate reader.

To further assess antibacterial efficacy, 6 µL of the incubated suspension was diluted with Mueller‐Hinton broth (MHB) to a final volume of 600 µL. A 100 µL sample of the resulting 10⁵‐fold diluted suspension was plated on agar and incubated at 37 °C for 12 h. Colony‐forming units (CFUs) were subsequently observed to confirm the bactericidal performance. All experiments were conducted in triplicate with independent repeats to ensure consistency for *E. coli* and *S. aureus*. The percentage of bacterial viability was calculated using the following formula:

(1) $$\begin{array}{ccc} & & \mathrm{Bacterial}\;\mathrm{viability}\;\left(\%\right)\\ & & =\frac{\mathrm{OD}\;\mathrm{of}\;\mathrm{treated}\;\mathrm{group}-\mathrm{OD}\;\mathrm{of}\;\mathrm{positive}\;\mathrm{control}\;\mathrm{group}}{\mathrm{OD}\;\mathrm{of}\;\mathrm{blank}\;\mathrm{control}-\mathrm{OD}\;\mathrm{of}\;\mathrm{positive}\;\mathrm{control}\;\mathrm{group}}\;\times 100\% \end{array}$$

### Demonstration of Applications

EPL‐*g*‐O3HT‐1 films (10 × 10 mm) were prepared using the drop‐cast method, consistent with the process utilized for thin films in the degradation experiments. Two films were placed on the upper forearm of a volunteer, spaced ≈2 cm apart, and functioned as active electrodes. Copper wires connected the films to an EMG sensor (Two‐channel EMG sensor, Sichiray), with medical transparent tape securing thin films and wires in place. The tape also served as a dielectric layer to prevent direct contact between the wires and the skin, thus avoiding potential short circuits. A reference electrode (Sichiray) was positioned on the elbow, a non‐active muscle, to work as the ground electrode. The EMG sensor was wirelessly connected to the computer software via Bluetooth for real‐time monitoring and recording of EMG signals. EMG signals from the forearm muscle were recorded during several repeated activities involving repeated muscle contraction and release motions: raising the forearm while keeping the hand open, raising the forearm with fist‐clenching at the end of the motion, and sliding the arm on a table while maintaining a consistent angle between the arms. All EMG demonstrations involving human participation were performed solely by the first author X.S. The written informed consent was obtained prior to the research and publication of the related data and images.

A 5 mL THF solution of EPL‐*g*‐O3HT‐1 (5 mg mL^−1^) was spin‐coated to form the OECT channel between the laser‐scribed graphene source and drain electrodes. The preparation of laser‐scribed graphene adopted the previously optimized parameters:^[^ ^41^ ^]^ a laser speed of 0.45 cm s^−1^, a laser pulse per inch (PPI) of 1000, and a laser power of 2.7 W. An Ag/AgCl electrode (versus 3 M NaCl) was utilized as the gate electrode. The EPL‐*g*‐O3HT‐1 channel had dimensions of 5 mm in length and 1 mm in width. Transfer curves (V_GS_ versus I_DS_) of the EPL‐*g*‐O3HT‐1‐based OECT were measured under different gate voltages using the LinkZill TruEbox TFT‐IV instrument. V_DS_ versus I_DS_ curves were obtained at various fixed gate potentials. The measurements were conducted using two different electrolytes: PBS buffer and LiClO_4_ in acetonitrile. Further experimental details were provided in Supporting Information.

### Statistical Analysis

Results were presented as mean ± standard deviation (SD). Error bars in the graphs represent the SD. Statistical significance for the bacterial viability was determined using an unpaired t‐test performed in GraphPad Prism 8.

## Conflict of Interest

The authors declare no conflict of interest.

## Author Contributions

X.S. conducted all experiments and prepared the initial draft. E.W.C.C., F.R.S., B.Z., J.Y., and K.M. contributed to the copolymer synthesis, antibacterial testing, OECT demonstrations and biocompatibility evaluation, and OECT performance improvement, respectively. V.S. supervised and provided financial support for the antibacterial tests. S.T. supervised the OECT performance improvement experiments. D.B. oversaw conceptualization, methodology, writing review and supervision. J.T.‐S. was responsible for conceptualization, methodology, writing review, supervision and financial support.

## Supporting information



Supporting Information

## Data Availability

The data that support the findings of this study are available from the corresponding author upon reasonable request.
