# Supplementary material for: ε‐Poly‐L‐lysine‐graft‐oligo(3‐hexylthiophene) Copolymers as Antibacterial and Biodegradable Polymer Electronics
Source: Adv Sci (Weinh). 2025 May 28;12(33):e01726. doi: 10.1002/advs.202501726 (PMC12412614; doi:10.1002/advs.202501726)
Supplement: Supplementary file 1 — Supporting Information [file ADVS-12-e01726-s001.docx]

**Supporting Information**

**ε-Poly-L-lysine-graft-oligo(3-hexylthiophene) Copolymers as Antibacterial and Biodegradable Polymer Electronics**

*Xin Sun,^1,2^ Eddie Wai Chi Chan,^1,2^ Fathumma Rizana Shiraz,^2,3^ Bicheng Zhu,^1,2^ Jingwen Yang,^1,2^ Katharina Matura,^4^ Viji Sarojini,^2,3^ Serpil Tekoglu,^4^ David Barker^1,2^ and Jadranka Travas-Sejdic^1,2*^*

^1^ Centre for Innovative Materials for Health, School of Chemical Sciences, The University of Auckland, Auckland 1010, New Zealand

^2^ MacDiarmid Institute for Advanced Materials and Nanotechnology, Wellington 6140, New Zealand

^3^ School of Chemical Sciences and The Centre for Green Chemical Science, University of Auckland, Auckland 1142, New Zealand

^4^ Linz Institute for Solar Cells and Institute of Physical Chemistry, Johannes Kepler University, Altenberger Strasse 69, Linz, Austria

* Corresponding author: Jadranka Travas-Sejdic, Email: [j.travas-sejdic@auckland.ac.nz](mailto:j.travas-sejdic@auckland.ac.nz)

## **Materials**

2,5-Dibromo-3-hexylthiophene and [1,3-bis(diphenylphosphino)propane]dichloronickel(II) (Ni(dppp)Cl2) were obtained from AK Scientific and used without further purification. Isopropyl magnesium chloride (*^i^*PrMgCl) and sodium azide were bought from Sigma Aldrich and used as received. 2-Bromo-5-(5-bromopentyl)-3-hexylthiophene was synthesized according to our previously published paper.[1] N-Succinimidyl 4-[(5-Aza-3,4:7,8-dibenzocyclooct-1-yne)-5-yl]-4-oxobutyrate (DBCO-NHS) was obtained from Accela ChemBio. ɛ-Poly-L-lysine hydrochloride (MW<5000) was obtained from Meryer. Trypsin from bovine pancreas (Type I, ~10,000 BAEE units per mg protein) was purchased from Merck and used as received.

## **Synthesis and characterisations of O3HT end-capped with NHS ester group (O3HT-NHS)**

- 1. **Synthesis of oligo-3-hexylthiophene** **with azide end-capped (O3HT-N_3_) 6**

To prepare the Grignard reagent intermediate **2** (Scheme S1), a solution of 2,5-dibromo-3-hexylthiophene **1** (1500 mg, 4.60 mmol, 30 eq.) in THF (15 mL) was cooled to 0 °C under nitrogen. Then, 2 M *^i^*PrMgCl in hexanes (30 eq.) was added, and the mixture was stirred for 30 min to obtain Grignard reagent intermediate **2** solution. This solution was transferred to another solution of Ni(dppp)Cl₂ (83.15 mg, 0.155 mmol, 1 eq.) in THF (5 mL), also cooled to 0°C under nitrogen atmosphere. The ratio of 2,5-dibromo-3-hexylthiophene **1** to catalyst Ni(dppp)Cl₂ was controlled to achieve the oligomer length (n) of approximately 30. The reaction was stirred for 10 min to form oligomers of 3-hexylthiophene **3**.

Separately, a solution of 2-bromo-5-(5-bromopentyl)-3-hexylthiophene **4** (150 mg, 0.385 mmol, 2.5 eq.) in THF (2.5 mL) was cooled to 0 °C under nitrogen. Two equivalents of *^i^*PrMgCl were added and the mixture was stirred for 30 min, forming the capping group intermediate **5**. This solution of **5** was then combined with the oligomer mixture **3** and stirred for another 10 min at 0 °C. The reaction was quenched by exposing it to air. The product was obtained by precipitation with methanol (300 mL), followed by centrifugation and washing with methanol and acetone. Bromide-capped O3HT (O3HT-Br) was obtained in 60% yield as a dark orange solid.

**Scheme S1. Synthesis of oligo(3-hexylthiophene) (O3HT) with azide end-capped 6.** 2,5-Dibromo-3-hexylthiophene **1** was transformed into the Grignard reagent **2**, which was subsequently oligomerized using controlled Grignard metathesis to produce oligomer **3**. Separately, 2-bromo-5-(5-bromopentyl)-3-hexylthiophene **4** was converted into arylmagnesium halide **5** and was employed to get the bromide capping group and stop the oligomerisation. The bromide capping group was subsequently converted to an azide group using sodium azide, yielding the azide-functionalized oligomer **6**.

A solution of bromide-capped O3HT (O3HT-Br) (400 mg, 0.0888 mmol, 1 eq.) in THF (10 mL) was combined with NaN_3_ (5.77 mg, 0.0888 mmol, 1 eq.) and stirred at room temperature overnight. The resulting azido oligomers were precipitated by adding methanol (200 mL), followed by centrifugation. The product was washed with methanol and water to yield azide-functionalized oligomers **6** (O3HT-N_3_**)** in 90% yield as a dark red solid.

- 1. **Synthesis of O3HT end-capped with NHS ester group 7 (O3HT-NHS)**

**Scheme S2.** The azide capping group of O3HT-N_3_ **6** was subsequently converted to the NHS group using DBCO-NHS linker, yielding the NHS-functionalized oligomer **7**.

To a solution of O3HT-N_3_ **6** (350 mg, 0.080 mmol, 1 eq.) in dichloromethane (DCM, 10 mL) was added DBCO-NHS (31.39 mg, 0.078 mmol, 1 eq.) and the mixture stirred at room temperature overnight. After this time, the crude product was precipitated with methanol (200 mL). The precipitate was collected through centrifugation and washed multiple times with methanol, water and acetone to remove residual reactants, giving NHS-functionalized O3HT oligomers **7** (O3HT-NHS) with a 93% yield (Scheme S2) as a dark red solid.

- 1. **Fourier transform-infrared (FTIR) spectroscopy of O3HT-Br, O3HT-N_3_ and O3HT-NHS**

FTIR spectroscopy was utilized to verify the successful conversion of functional groups during the synthesis of O3HT-NHS. As illustrated in Figure S17, after reacting with NaN_3_, the spectrum of O3HT-N_3_ exhibited a new peak at 2091 cm⁻¹ that corresponds to the azide (N_3_) group, while the characteristic C-Br peak disappeared, confirming the successful transformation of bromide to azide groups in O3HT. Subsequently, in the spectrum of O3HT-NHS, the disappearance of the N_3_ peak and the presence of the C=O peak at 1640 cm⁻¹, characteristic of the NHS group, indicated the successful conversion of azide groups to NHS ester groups.

## **Characterisations of EPL-*g*-O3HTs**

### **GPC**

All GPC samples were analysed at a temperature of 50 °C, using THF as the mobile phase at a flow rate of 1 mL min^-1^. Data processing was carried out using Lab Solution software. Calibration was performed using 5-point polystyrene standards (Shodex Standard), with molecular weights of 4880, 6540, 10300, 13000, and 22300 g mol^-1^, respectively.

### **Cyclic voltammetry**

In the CV experiments, gold electrodes were initially polished with BASi alumina (0.05 µm) on a polishing pad, followed by thorough rinsing with deionized water. This was succeeded by sonication in ethanol and deionized water for 5 min each. The gold electrodes were further subjected to electrochemical cleaning by scanning from -0.2 V to 1.6 V in 1 M H_2_SO_4_​ for 12 cycles, after which they were rinsed with deionized water and dried with nitrogen gas. After electrode preparation, 15 μL of the polymer solution (5 mg mL^-1^) was carefully deposited onto the gold electrode surface and subsequently dried under vacuum for 30 min

. Cyclic voltammograms of EPL-*g*-O3HTs were recorded by potential cycling from 0 to 1.2 V (*vs.* Ag/AgCl) in MeCN containing 0.2 M lithium perchlorate at varying scan rates (25 to 125 mV s^-1^).

### **OECT device fabrication**

To further achieve high signal amplification in EPL-*g*-O3HT-1-based OECTs, another OECT device was fabricated as follows. The source-drain electrode pattern (channel length: *L* = 60 µm, channel width *W* = 2 mm) was prepared by fixing cleaned glass substrates onto a shadow mask. 10 nm chromium (Cr) and 100 nm gold (Au) were deposited sequentially by thermal evaporation. To deposit a thicker channel layer (approx. 5 μm thick), EPL-*g*-O3HT-1 solution (7 mg ml^-1^ in THF) was drop-cast as channel polymer. The area around the channel was cleaned by cotton swabs. To complete the devices, a laser-cut polymer well of 3M^TM^ VHB^TM^ Tape (acrylic adhesive with a conformable acrylic foam core) was fixed on the substrates to secure the electrolyte. For the device characterization, 20 µL of LiClO_4_ electrolyte in acetonitrile (0.1 M, pH 7.4) was used, and a silver/silver chloride (Ag/AgCl) electrode was employed as a non-polarizable gate electrode. The steady-state current-voltage measurements were performed under ambient conditions using an Agilent model E5273A.

**Figure S1.** Calibration graph of O3HT-NHS absorbance in THF *vs*. concentration.


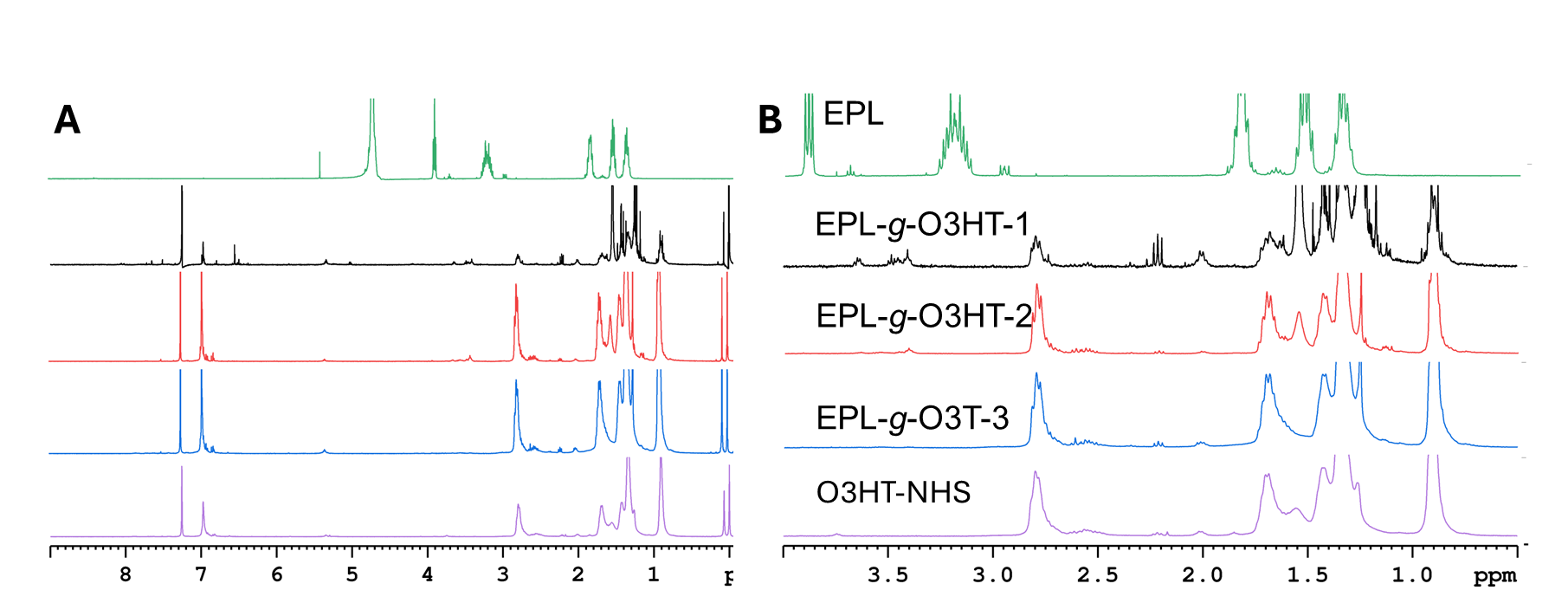


**Figure S2.** Full range (A) and zoomed-in (B) ^1^H NMR spectra of pristine ɛ-poly-L-lysine (EPL), O3HT-NHS and EPL-*g*-O3HTs copolymers.


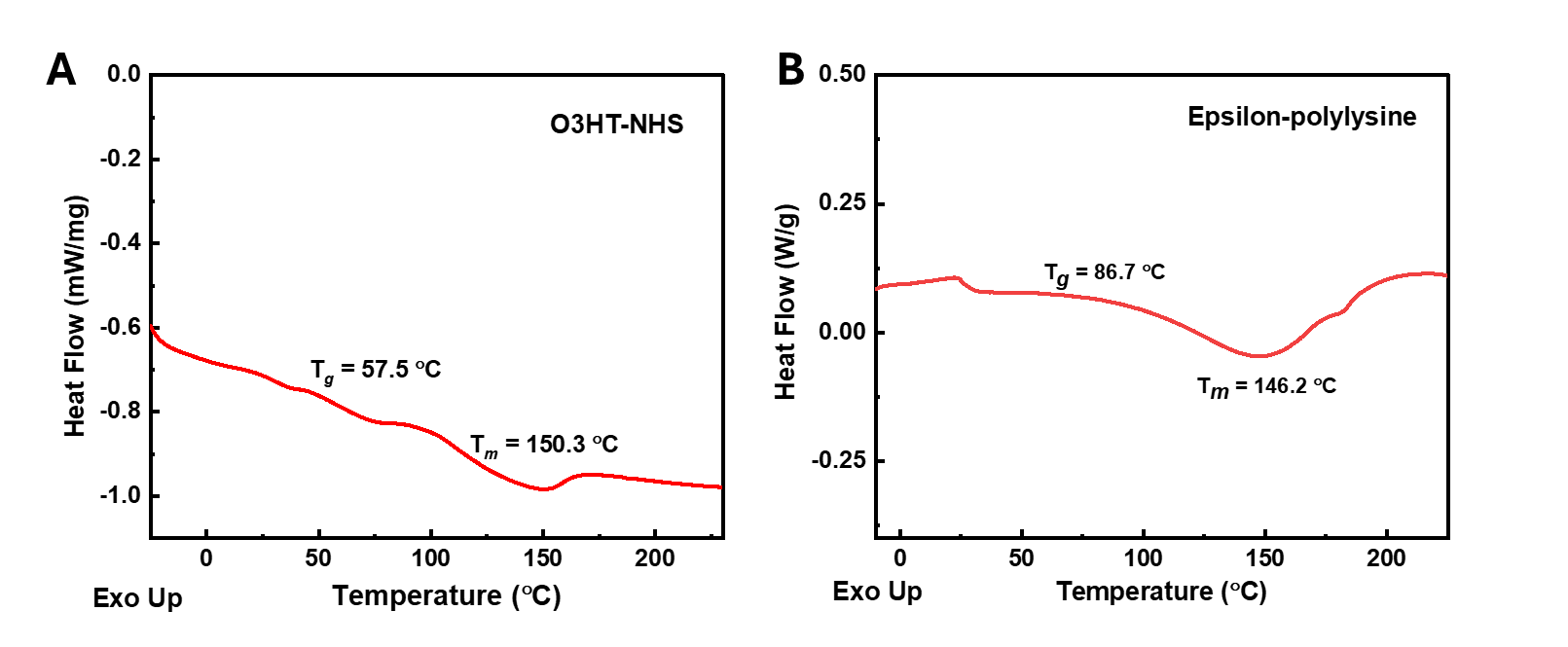


## **Figure S3.** DSC thermograms of (A) O3HT-NHS and (B) pristine ɛ-poly-L-lysine

## **Figure S4.** Derivation of weight change in TGA curves of pristine ɛ-poly-L-lysine, O3HT-NHS, and EPL-*g*-O3HTs copolymers.

## **Figure S5.** The Raman spectrum of pristine ɛ-poly-L-lysine.

|  | **N** | **S** | **S/N (wt. % / wt. %）** |
| --- | --- | --- | --- |
| **EPL-*g*-O3HT-1** | 1.02 | 9.576 | 9.4 |
| **EPL-*g*-O3HT-2** | 0.97 | 11.402 | 11.8 |
| **EPL-*g*-O3HT-3** | 0.62 | 15.008 | 24.2 |
| **O3HT-NHS** | 0.39 | 15.741 | 40.4 |

## **Table S1.** Elemental analysis results of EPL-*g*-O3HTs and O3HT-NHS.

|  | **Neutral (I*_C-C_*/I*_C=C_*)** | **Doped (I*_C-C_*/I*_C=C_*)** |
| --- | --- | --- |
| **O3HT-NHS** | 0.33 | 0.60 |
| **EPL-*g*-O3HT-1** | 0.36 | 0.76 |
| **EPL-*g*-O3HT-2** | 0.39 | 0.75 |
| **EPL-*g*-O3HT-3** | 0.41 | 0.53 |

## **Table S2.** The I_C-C_/I_C=C_ ratio in the Raman spectra of pristine ɛ-poly-L-lysine, O3HT-NHS, and EPL-*g*-O3HTs copolymers in neutral and doped state.

|  | **Conductivity (S/m)** |
| --- | --- |
| **O3HT-NHS** | 19.23 ± 0.30 |
| **EPL-*g*-O3HT-1** | 1.13 ± 0.03 |
| **EPL-*g*-O3HT-2** | 1.36 ± 0.02 |
| **EPL-*g*-O3HT-3** | 1.98 ± 0.05 |

## **Table S3.** Thin film conductivity measured by four-point probe.


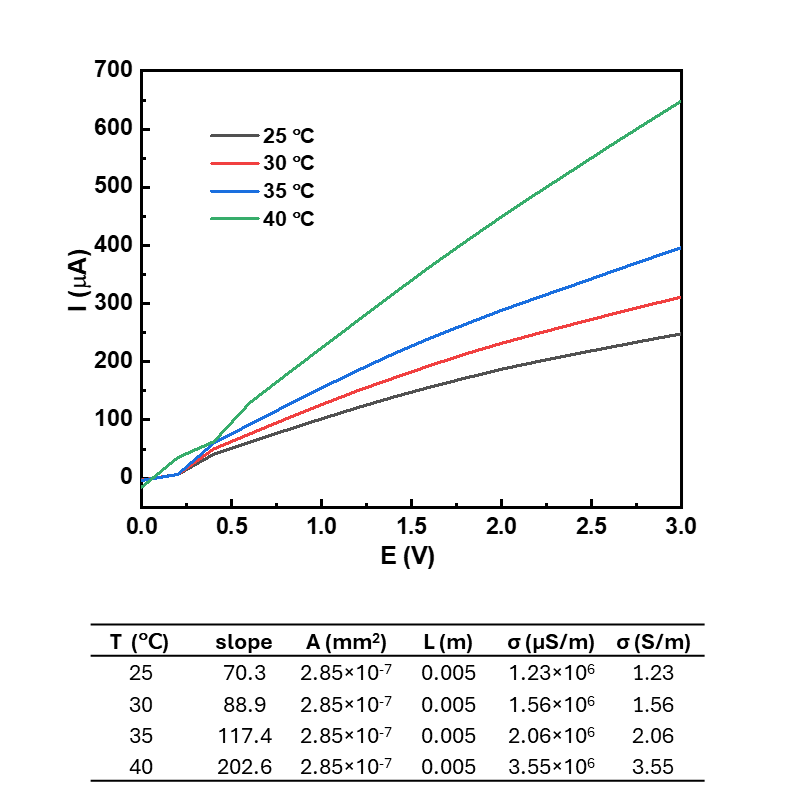


## **Figure S6.** I-V curves (from 0 V to 3 V) of EPL-*g*-O3HT-1 thin films at temperatures between room temperature (25 °C) and 40 °C.

## **
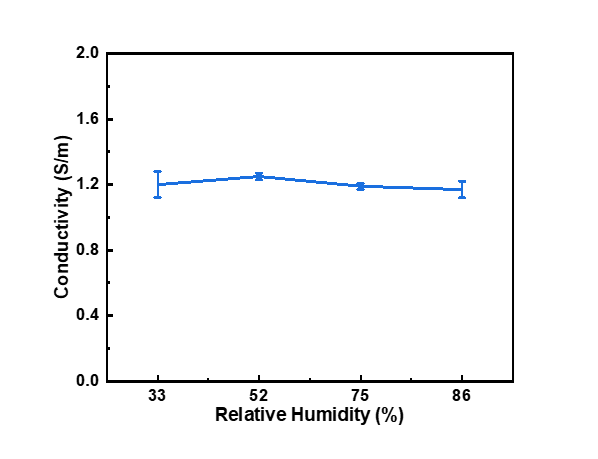
Figure S7**. Conductivity of EPL*-g*-O3HT-1 thin films measure at room temperature in varying relative humidities using four-point probe.


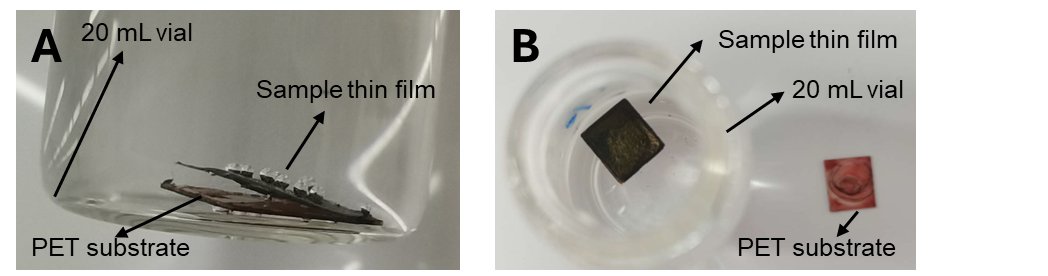


## **Figure S8.** Photographs illustrating the thin film fabrication process using dextran as a sacrificial layer. (A) Close-up view of a thin film during the early stage of film delamination. After drop-casting the polymer solution onto a dried dextran layer on a PET substrate (10 mm × 10 mm), the substrate was immersed in PBS at 60 °C. As the dextran layer dissolves, the thin film begins to peel off from the substrate. (B) Top view after 4–6 hours, showing that the thin film becomes fully detached, floating on the PBS surface. The image on the right shows the PET substrate that previously underneath the film, confirming complete release of the film. Further details are provided in Section 4.3 of the Experimental section.


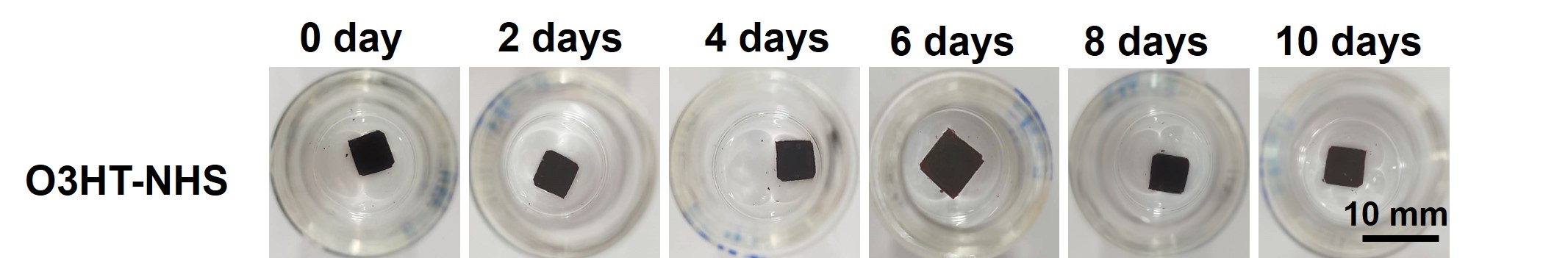


## **Figure S9.** Optical images of O3HT-NHS thin films over a 12-days of enzymatic degradation in PBS.


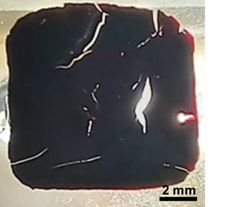


## **Figure S10.** The optical image of the EPL-*g*-O3HT-3 thin film after 12 days of enzymatic degradation in PBS, with visible cracks on it.

**
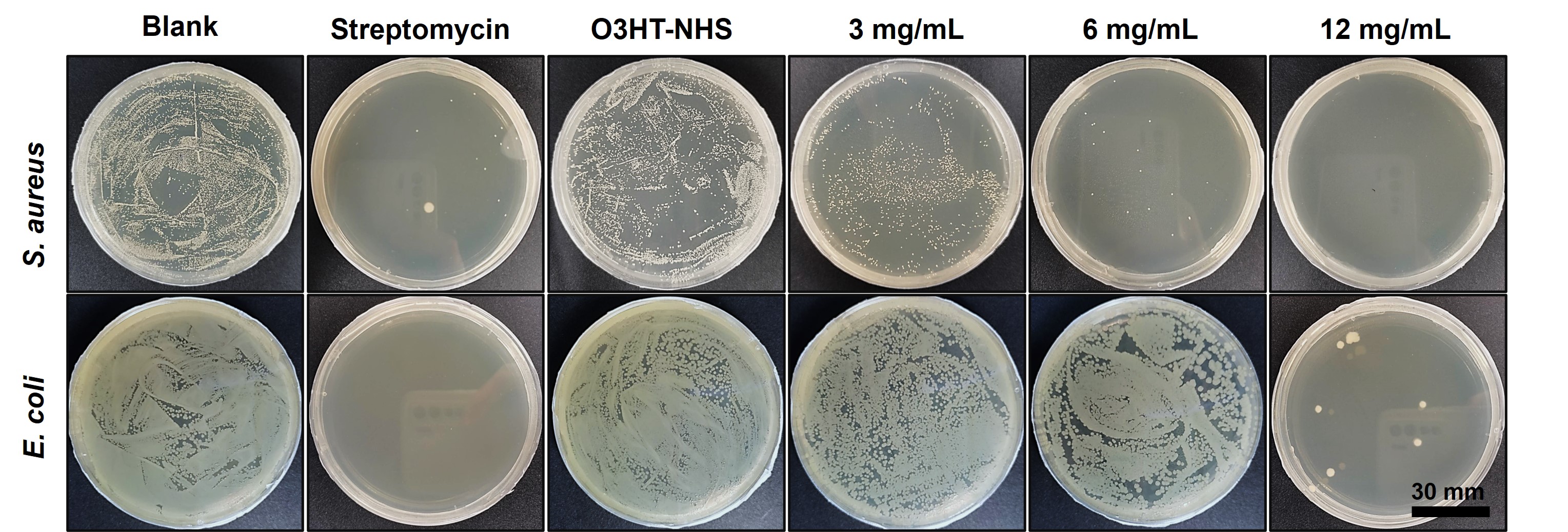
**

## **Figure S11.** Photographs of the survival bacterial colonies on agar plates to demonstrate in vitro antibacterial activities of O3HT-NHS and EPL-*g*-O3HTs copolymers against *E. coli* and *S. aureus*, respectively. The positive control groups, treated with streptomycin, are included for comparison.

| **Polymer** | **Fabrication Strategy** | **Electrical Conductivity** | **Degradation Performance** | **Ref** |
| --- | --- | --- | --- | --- |
| EPL-*graft*-O3HTs | Degradable  backbone | 1.13 ± 0.03 S/m | Enzymatic degradation in 12 days | This work |
| Gelatin-*graft*-poly(3-  hexylthiophene) | Degradable  backbone | (1.65 ± 0.02) × 10^-5^ S/m | Enzymatic degradation in 5 days | [2] |
| P(CL-*co*-AVL)-*g*-O3HT | Degradable  backbone | 5.6 × 10^-1^ S/m | Acid, base and  enzymatic  degradation | [1] |
| Heparin-G-PEDOT | Degradable  backbone | 1 × 10^−3^ S/m | Hydrogel in vivo degradation | [3] |
| PEDOT-graft-PCL | Degradable  backbone | 7.07 × 10^−2^ S/m | Not investigated | [4] |
| PEDOT-graft-PLA | Degradable  backbone | 300 ± 24.1 µS/cm | Not investigated | [5] |
| Poly(naphthalene diimideco-(E,E)-N,N’1,4-phenylenebis[1-(2-thienyl)methanimine]) (PNDIT2/IM-f) | Degradable  semiconductor | Electron  mobility (μe)  0.04 cm2 V−1  s−1 | Acid hydrolysis | [6] |
| PPy-b-PCL copolymers | Degradable block copolymer | 2.8 × 10^−2^ S/m | 0.5MNaOH solution in 14 days | [7] |
| PEDOT-co-PDLLA copolymer | Degradable block copolymer | 5.35 × 10^-3^ S/m | 35 days of enzymatic degradation | [8] |
| Water-soluble transient  conjugated polymer based  on polypyrrole (PPY) | Soluble copolymer | Capacitance value 73 mF/g | Water soluble | [9] |
| PEDOT modified with COOH | Soluble copolymer | Charge transport resistance (*R*_ct_) of 425 Ω | complete dissolution after 7 days at pH 7.4 | [10] |
| PEDOT/PLA | Composite | Sheet resistance: 400 Ω/sq | Not investigated | [11] |
| PEDOT:PSS/montmorillonite | Composite | 8 to 16 S/cm | Ingestion by  superworms | [12] |
| PCL/PANI | Composite | 2.5 × 10^−2^ S/m | Not investigated | [13] |

## **Table S4.** Conducting polymer-based biodegradable electronic materials.


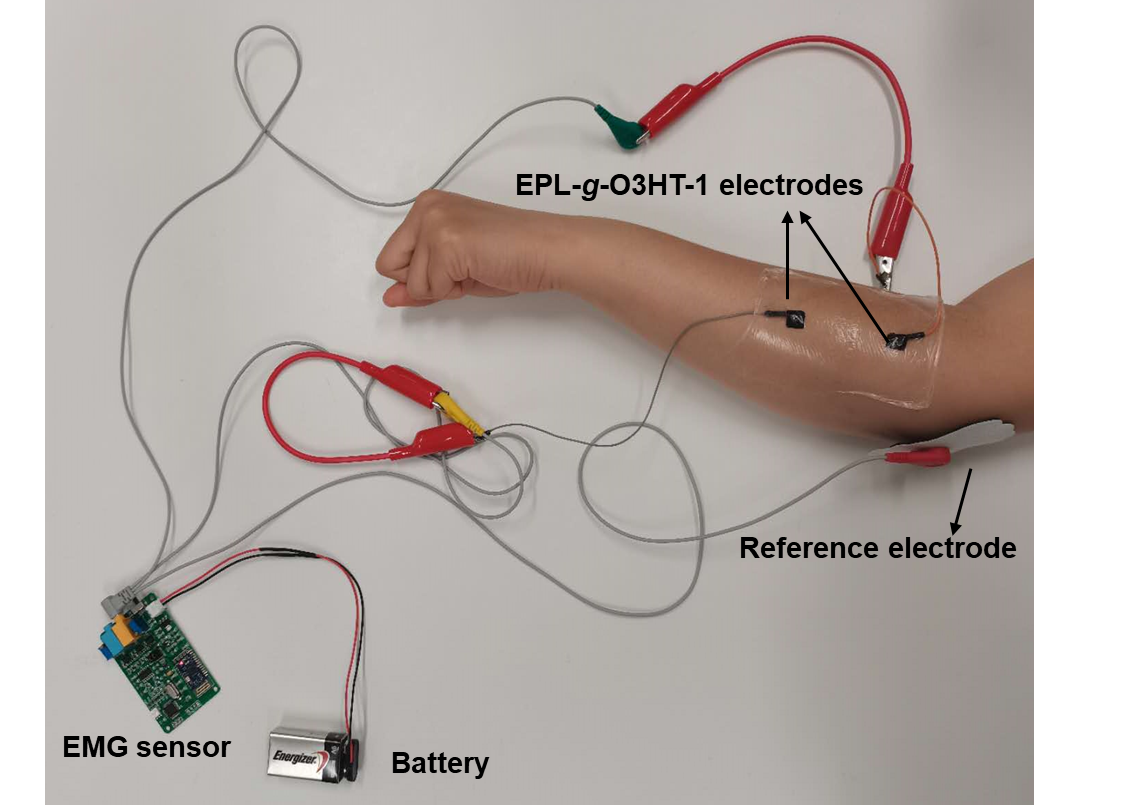


## **Figure S12.** Photograph of EPL-*g*-O3HT-1 thin films used as EMG sensor electrodes. The sensor communicates with a computer *via* Bluetooth, and the data are monitored using dedicated software.

## **Figure S13.** EMG signals of the forearm muscle were recorded as the volunteer slide their arm outward along a desk, keeping the angle between the arms constant.


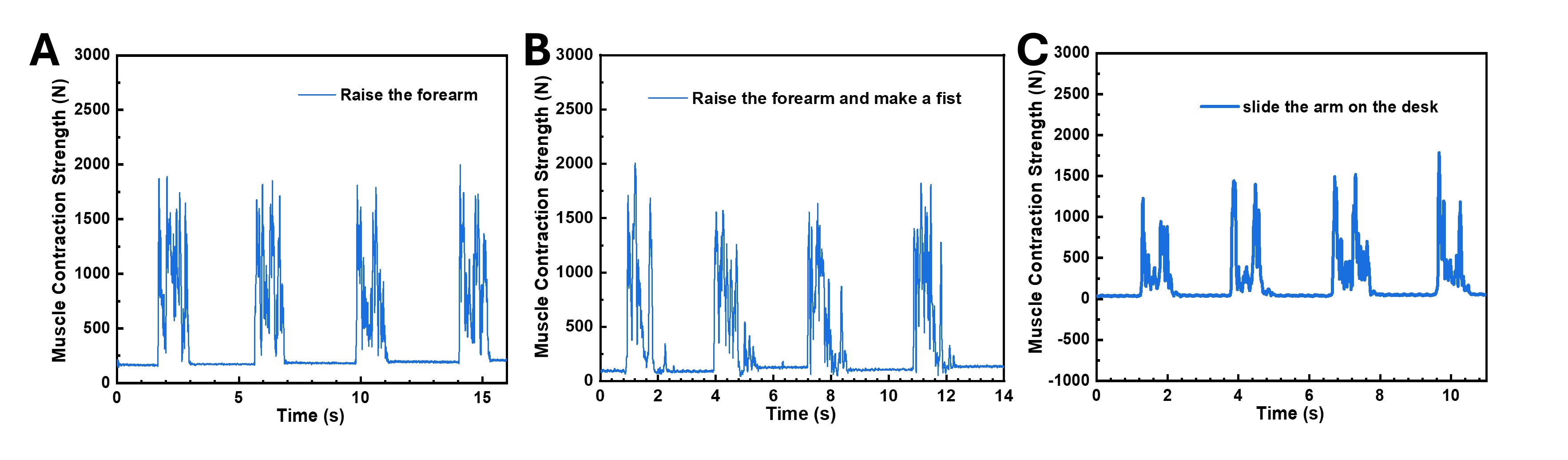


## **Figure S14.** Electromyography (EMG) signals recorded from the forearm muscle while the volunteer performed various actions. The Y-axis represents muscle contraction strength, derived from filtered and converted raw EMG data.


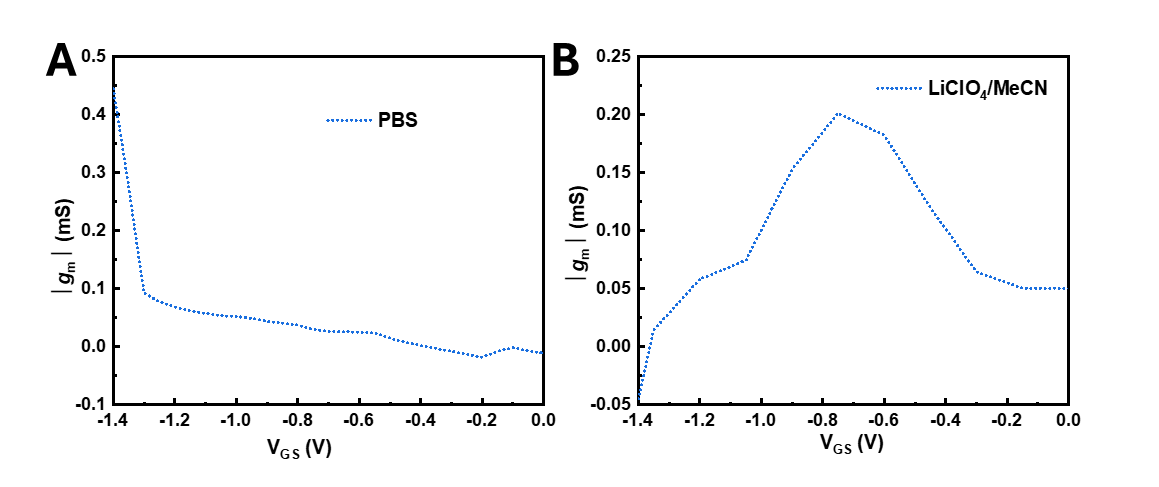


## **Figure S15.** Transconductance of OECTs when using EPL-*g*-O3HT-1 as channel material in different electrolytes.

**
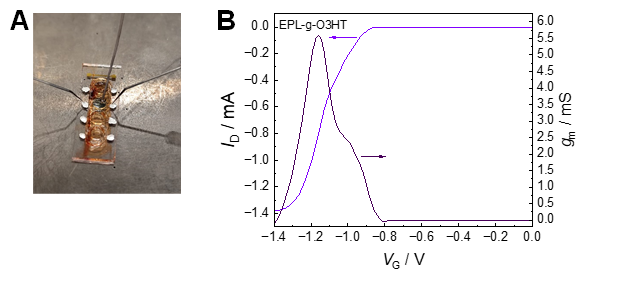
**

## **Figure S16.**(A) EPL-*g*-O3HT-1 OECT under operation and (B) transfer characteristic and corresponding transconductance value (*g*_m_) of OECT with 0.1 M LiClO_4_ electrolyte in acetonitrile (drain voltage *V*_DS_= -0.7 V, the channel *W* = 2 mm, *L* = 60 µm, and the film thickness (*d)=* ~ 5 μm).

## **Figure S17.** FTIR spectra of O3HT-Br, O3HT-N_3_, and O3HT-NHS.


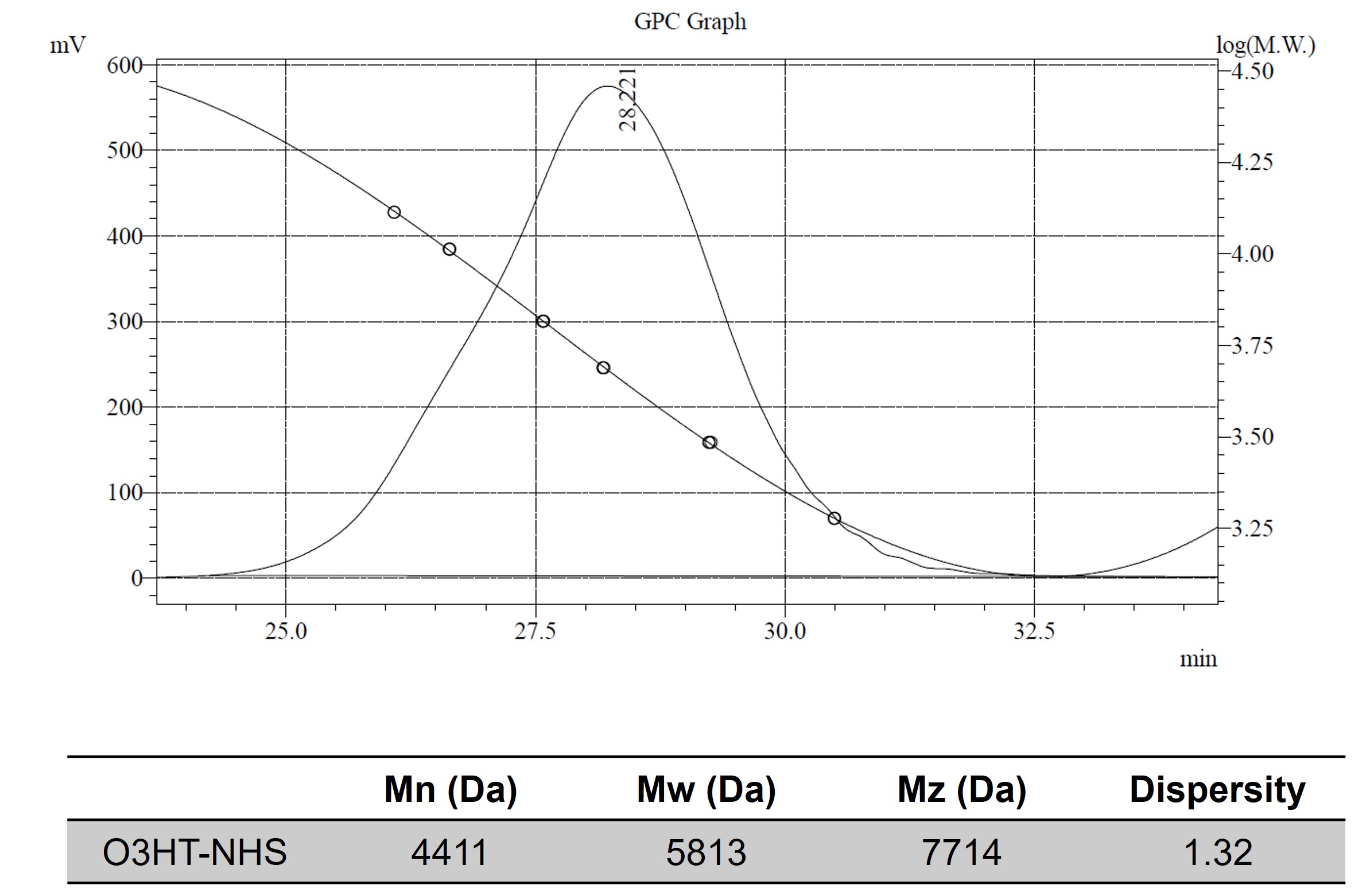


## **Figure S18.** GPC result of prepared O3HT-NHS.

## **Reference**

[1] E. W. C. Chan, X. Sun, Y. Uda, B. Zhu, D. Barker, J. Travas-Sejdic, *J. Mater. Chem. C* **2024**, *12*, 11157.

[2] X. Sun, E. W. C. Chan, Q. Chen, N. Kirby, J. Yang, J. P. Mata, R. L. Kingston, D. Barker, L. Domigan, J. Travas-Sejdic, *ACS Appl. Mater. Interfaces* **2024**, *16*, 23872.

[3] D. Hachim, O. Hernández‐Cruz, J. E. Foote, R. Wang, M. W. Delahaye, D. J. Stuckey, Z. Feng, J. P. Wojciechowski, L. C. Salter, J. Lin, *Advanced Healthcare Materials* **2025**, 2403995.

[4] A. Dominguez-Alfaro, M. Criado-Gonzalez, E. Gabirondo, H. Lasa-Fernández, J. L. Olmedo-Martínez, N. Casado, N. Alegret, A. J. Müller, H. Sardon, A. Vallejo-Illarramendi, D. Mecerreyes, *Polym. Chem.* **2022**, *13*, 109.

[5] A. Dominguez-Alfaro, E. Gabirondo, N. Alegret, C. M. De León-Almazán, R. Hernandez, A. Vallejo-Illarramendi, M. Prato, D. Mecerreyes, *Macromol. Rapid Commun.* **2021**, *42*, 2100100.

[6] H. Park, Y. Kim, D. Kim, S. Lee, F. S. Kim, B. J. Kim, *Adv. Funct. Mater.* **2022**, *32*, 2106977.

[7] S. Vijayavenkataraman, S. Kannan, T. Cao, J. Y. Fuh, G. Sriram, W. F. Lu, *Frontiers in bioengineering and biotechnology* **2019**, *7*, 266.

[8] A. C. da Silva, A. T. S. Semeano, A. H. B. Dourado, H. Ulrich, S. I. Cordoba de Torresi, *ACS Omega* **2018**, *3*, 5593.

[9] J. Moon, V. Diaz, D. Patel, R. Underwood, R. Warren, *Org. Electron.* **2022**, *101*, 106412.

[10] X. Jia, X. Ma, L. Zhao, M. Xin, Y. Hao, P. Sun, C. Wang, D. Chao, F. Liu, C. Wang, *Chemical Science* **2023**, *14*, 2123.

[11] Y. Won, J. J. Lee, J. Shin, M. Lee, S. Kim, S. Gandla, *ACS Sens.* **2021**, *6*, 967.

[12] S. Lee, Y. Hong, B. S. Shim, *Adv. Sustain. Syst.* **2022**, *6*, 2100056.

[13] A. Wibowo, C. Vyas, G. Cooper, F. Qulub, R. Suratman, A. I. Mahyuddin, T. Dirgantara, P. Bartolo, *Materials* **2020**, *13*, 512.
